# Battery-free, wireless, and electricity-driven soft swimmer for water quality and virus monitoring

**DOI:** 10.1126/sciadv.adk6301

**Published:** 2024-01-10

**Authors:** Dengfeng Li, Jingkun Zhou, Zichen Zhao, Xingcan Huang, Hu Li, Qing’ao Qu, Changfei Zhou, Kuanming Yao, Yanting Liu, Mengge Wu, Jingyou Su, Rui Shi, Ya Huang, Jingjing Wang, Zongwen Zhang, Yiming Liu, Zhan Gao, Wooyoung Park, Huiling Jia, Xu Guo, Jiachen Zhang, Pakpong Chirarattananon, Lingqian Chang, Zhaoqian Xie, Xinge Yu

**Affiliations:** ^1^ Department of Biomedical Engineering, City University of Hong Kong, Hong Kong SAR 999077, China.; ^2^ Hong Kong Centre for Cerebro-Cardiovascular Health Engineering (COCHE), Hong Kong SAR 999077, China.; ^3^ State Key Laboratory of Structural Analysis, Optimization and CAE Software for Industrial Equipment, Dalian University of Technology, Dalian 116024, China.; ^4^ Department of Engineering Mechanics, Dalian University of Technology, Dalian 116024, China.; ^5^ Beijing Advanced Innovation Center for Biomedical Engineering, School of Biological Science and Medical Engineering, Beihang University, Beijing 100083, China.; ^6^ School of Information and Communication Engineering, Dalian University of Technology, Dalian 116024, China.; ^7^ Ningbo Institute of Dalian University of Technology, Ningbo 315016, China.; ^8^ School of Biomedical Engineering, Research and Engineering Center of Biomedical Materials, Anhui Medical University, Hefei 230032, China.; ^9^ DUT-BSU Joint Institute, Dalian University of Technology, Dalian 116024, China.; ^10^ City University of Hong Kong Shenzhen Research Institute, Shenzhen 518057, China.

## Abstract

Miniaturized mobile electronic system is an effective candidate for in situ exploration of confined spaces. However, realizing such system still faces challenges in powering issue, untethered mobility, wireless data acquisition, sensing versatility, and integration in small scales. Here, we report a battery-free, wireless, and miniaturized soft electromagnetic swimmer (SES) electronic system that achieves multiple monitoring capability in confined water environments. Through radio frequency powering, the battery-free SES system demonstrates untethered motions in confined spaces with considerable moving speed under resonance. This system adopts soft electronic technologies to integrate thin multifunctional bio/chemical sensors and wireless data acquisition module, and performs real-time water quality and virus contamination detection with demonstrated promising limits of detection and high sensitivity. All sensing data are transmitted synchronously and displayed on a smartphone graphical user interface via near-field communication. Overall, this wireless smart system demonstrates broad potential for confined space exploration, ranging from pathogen detection to pollution investigation.

## Teaser

A battery-free, wireless, and soft electromagnetic swimmer enables water quality and virus monitoring in confined environments.

## INTRODUCTION

In situ monitoring is critical for exploring confined spaces such as inside long domestic and industrial pipes or even the human body. For instance, monitoring water quality and pathogen contamination inside confined domestic water pipes could help protect public health by timely detecting pollution, such as avoiding diet-related diseases, and controlling the spread of viral infectious diseases (*1*, *2*), but these areas are not easily physically accessed by humans for manual in situ sampling. Although regular wastewater sampling can be conducted at the pipeline outlet, such terminal detection fails to trace the specific origin of contamination and lacks timeliness (*3*–*6*). One feasible solution is to develop a miniature multifunctional mobile electronic system that can move inside confined spaces for real-time in situ monitoring (*7*–*11*). However, critical barriers exist in designing such a system. Specifically, the entire system needs to operate fully wirelessly (*12*), as any wires entanglement will restrict its dexterous motion (*13*, *14*). Moreover, the sensing data must be extracted wirelessly, since repeatedly removing the system from confined spaces is inconvenient. Although magnetic soft robots show promise as such wireless mobile systems owing to their dexterous locomotion and adaptability in confined narrow spaces, their lack of detection capabilities limits their application in this monitoring scenario (*15*–*17*). Therefore, novel small soft mobile monitoring systems enabling untethered motion, multifunctional sensing, and wireless data acquisition have been highly sought after for monitoring confined spaces, yet remain as a long-standing challenge.

In confined spaces, wireless operation is a key issue, covering power supply, mobility, and data transmission (*18*). On the one hand, electricity remains the most efficient, stable, and preferred method for both powering mobile monitoring systems and measuring sensing signals (*19*, *20*). Previous studies mostly used batteries for wireless electrical actuation of mobile soft robotic devices. However, batteries pose many limitations including notable weight burden, limited operation life, and increased moving resistance (*21*). The use of batteries inevitably requires additional control systems, circuits, and even wireless charging modules (*22*). The notable increase in weight and size is not conducive to miniaturization and lightweight of the entire system. Thus, developing novel powering technologies with battery-free features will bring tremendous value to mobile electronic monitoring systems (*23*). On the other hand, Bluetooth is commonly used for wireless sensing signal acquisition but its continuous power consumption still relies on battery power (*24*, *25*). Given that, novel battery-free wireless data acquisition and analysis methods need to be introduced. Meanwhile, integrating as many sensors as possible based on various sensing principles such as electrical property changes (*26*), electrochemical potentials (*27*, *28*), or bio-specific effects (*29*) is also necessary to greatly improve the system’s perception of unknown environments using multiple indicators. On the basis of the above, meeting so many functional requirements inevitably poses great difficulty for miniaturization of the mobile electronic monitoring system.

In this article, we introduce a set of materials, structural designs, sensor developments, circuit layouts, and integration strategies to develop a battery-free wireless soft electromagnetic swimmer (SES) monitoring system that enables wireless in situ detection of temperature, chemical ions, and virus in confined water pipes. The thin, soft, and wireless design in actuation and data sensing modules contributes to the lightweight and miniaturization of the entire monitoring system, which guarantees its free motion and operation in small confined spaces. With radio frequency (RF) powering, the system harvests energy from external radio signals to achieve untethered locomotion without batteries. The resonant electromagnetic actuation design also enables SES to swim fast in water. At the same time, multifunctional physical, chemical, and biological sensors substantially enhance this SES system’s perception capabilities and monitoring applications. Battery-free near-field communication (NFC) for wireless data transmission ensures that the SES can send sensing data wirelessly without any cable connections. Overall, the system significantly enhances the efficacy and flexibility of mobile electronic monitoring system for environmental exploration without compromising performance in narrow confined spaces.

## RESULTS

### Concept and functions of the battery-free wireless SES monitoring system

**Fig. 1.**
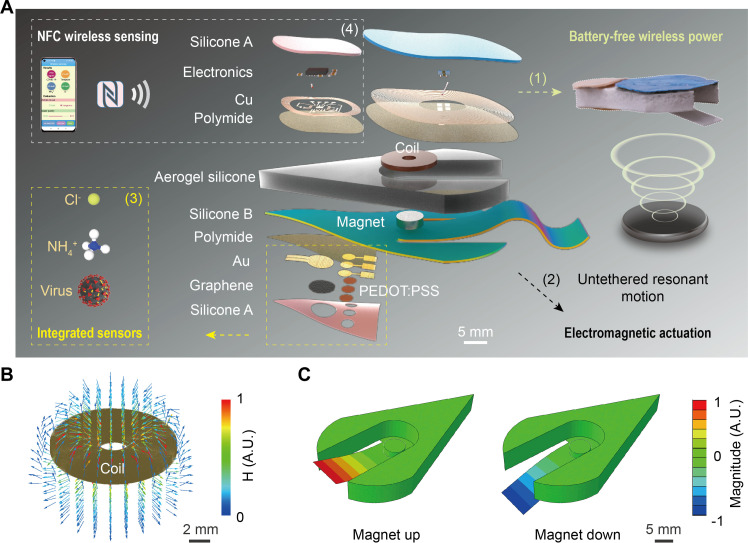
Architecture and functions of a battery-free wireless SES monitoring system. (**A**) Exploded-view schematic illustration of the system with four key modules, including (*1*) battery-free wireless RF powering, (*2*) electromagnetic actuation, (*3*) integrated sensors for ion and virus monitoring, and (*4*) NFC wireless sensing. (**B**) FEA-simulated magnetic field distribution of the electromagnetic actuation coil. (**C**) Vibration magnitude of the soft tail during electromagnetic actuation simulated by FEA. A.U., arbitrary units.

As illustrated in Fig. 1A, the battery-free wireless SES monitoring system is constructed with a multilayer stacked structure, comprising (*1*) a wireless power receiving module as an RF energy harvester containing a receiver antenna and paired electronics for rectification; (*2*) a soft tail connected to a floatable aerogel silicone support and an electromagnetic actuation system for mechanical propulsion; (*3*) an integrated sensor array as the detection module for measuring the temperature, ions, and virus; and (*4*) an NFC module for wireless sensor data acquisition and a smartphone graphical user interface (GUI) to display the data (fig. S1).

Enabled by the lightweight, soft, thin design with flexible electrode processing (fig. S2), the wireless power harvester module weighs only 1.1 g even with silicone encapsulation, much lighter than the battery-powered actuation module. The actuation coil, magnet, and electronic components all offer industrial-grade corrosion resistance. To protect the flexible electrode from corrosion, chemically stable polydimethylsiloxane (PDMS) was selected for encapsulation to meet the long-term use requirements in daily water environments (*30*). Then, by integrating a multifunctional thin-film sensor and an NFC module for wireless data reading, we have achieved miniaturization of the SES monitoring system with dimensions of 5.3 cm in length, 3.2 cm in width, and 5.3 g in weight (table S1). Under wireless powering, the electromagnetic swimmer periodically beats its tail fin to effectively swim in water (Fig. 1, B and C). Via wireless sensing and GUI display, the SES can enter confined locations for in situ monitoring. Thus, only the SES monitoring system and a smartphone are needed to easily acquire comprehensive information on physical, chemical, and biological indicators in water.

### Battery-free wireless power transfer

**Fig. 2.**
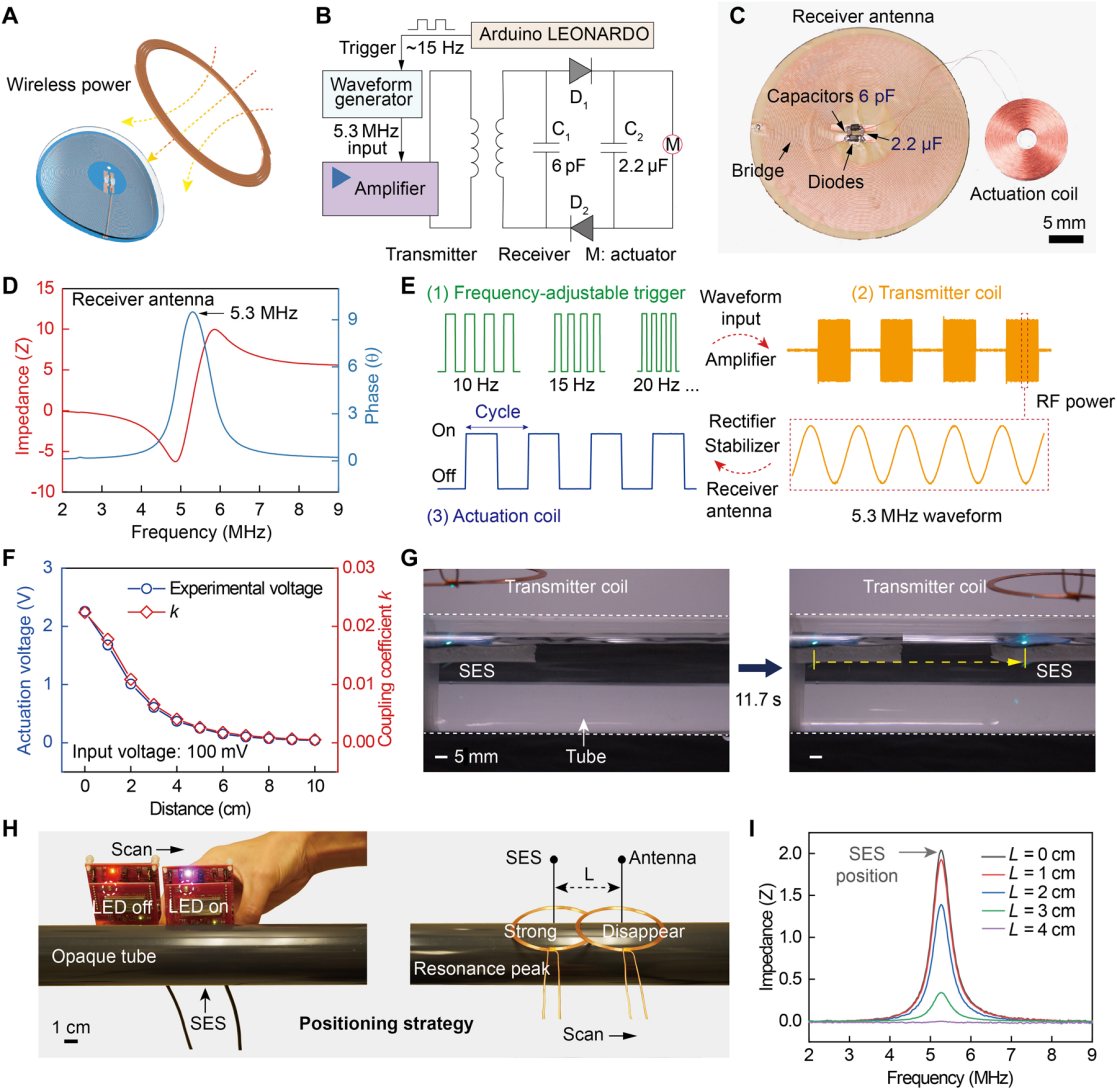
Battery-free wireless actuation strategies. (**A**) Schematic diagram of the wireless RF power. (**B**) Circuit diagram of the wireless actuation system. (**C**) Photograph of the actuation module including a receiver antenna and an actuator coil. (**D**) Matched resonant frequency testing between the transmitter coil and the receiver antenna. (**E**) Wireless actuation process: waveform input under a frequency-adjustable trigger, power amplification, wireless RF power, rectification, voltage stabilization, and square-wave voltage actuation. (**F**) Experimental actuation voltage on the actuation coil and simulated coupling coefficient *k* versus distance between the coaxial transmitter coil and receiver antenna. (**G**) Wirelessly actuating the SES to swim in a tube. (**H**) Positioning the SES system in opaque tubes by using an NFC transceiver or a resonant peak test coil. (**I**) Strongest resonant peak corresponding to the position of the SES system in opaque tubes.

Wireless actuation is necessary for untethered motion of the mobile electronic monitoring system in confined spaces. Compared to magnetic-, optical-, and pneumatic-based driven methods for soft robots (*31*–*33*), electrically driven robotic devices can be more precisely controlled, as they simply rely on electricity input instead of complex control systems. Batteries are the most used power supply method, but their size, along with supporting circuits, is still too large to enable the high integration needed for monitoring systems. Therefore, we introduce wireless RF technology to power the mobile SES monitoring system (Fig. 2). As a battery-free approach, it is greatly superior to batteries due to its features of ultra-thin, lightweight construction, elimination of charging needs, adjustable power, and facile motion control. As illustrated in Fig. 2A, only a transmitter coil is required to emit RF electromagnetic fields to the receiver antenna on the system for noncontact energy harvesting. As shown in the circuit design in Fig. 2 (B and C), energy is optimally transmitted to the receiver antenna at the matched resonant frequency of 5.3 MHz between the transmitter coil and receiver antenna (Fig. 2D). The measured power conversion efficiency from the transmitter coil to the coaxially aligned receiver antenna with a 0-cm distance is 1.6%. Notably, the resonant frequency only drifts 5% even under a large bending curvature of 0.1 mm^−1^, ensuring stable power supply during operation (fig. S3). Through a series of frequency-adjustable triggers, power amplification, wireless RF power, rectification, and voltage stabilization, the sinusoidal input voltage can be finally converted into a low-frequency square-wave actuation voltage to load onto the actuation coil for periodic electromagnetic oscillation (Fig. 2E).

To quantitatively characterize the electromagnetic actuation, we measured the actuation voltage across the actuation coil under wireless RF powering. At a coaxial distance of 0 cm between the transmitter coil and receiver antenna, the actuation voltage reaches 2.26 V under an input voltage of 100 mV. As the distance increases to 2 and 4 cm, the actuation voltage decreases to 1.01 and 0.37 V, respectively. Electromagnetic simulation of the wireless power transfer shows that the experimental actuation voltages match well with the simulated coupling coefficient $k(k=\frac{\Psi_{\text{RT}}}{I_{T}\sqrt{L_{R}L_{T}}})$ values (Fig. 2F). Ultimately, enabled by the above battery-free wireless power supply, this SES monitoring system can swim freely in a pipe, as demonstrated in Fig. 2G. Considering that most pipes are typically opaque, two strategies can be deployed to solve the positioning issue. As shown in Fig. 2H, an NFC transceiver (TRF7970A) or a coil was used to scan the opaque tube. Once the NFC transceiver lighted up (movie S1) or the strongest resonant peak appeared (Fig. 2I), the location of the SES device can be precisely determined. For real daily pipes such as polyvinyl chloride (PVC) in Fig. 2H, the RF energy collection and swimming motion were the same as those in the acrylic pipe. However, this swimmer is not suitable for use in metal pipes due to their strong electromagnetic shielding properties.

### Untethered motion in confined spaces

**Fig. 3.**
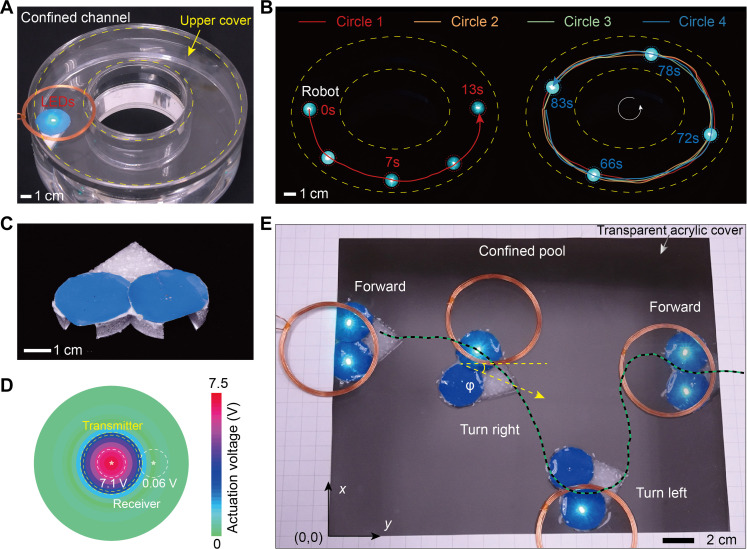
Untethered motion and steering in confined spaces. (**A**) SES located in a confined channel. (**B**) In darkness, the SES swims in circles around the confined channel untethered with LED tracking. (**C**) Top view of an upgraded directionally controllable SES with a symmetrical pair of receiver antennas and electromagnetic oscillation units. (**D**) Distribution of the actuation voltage on the actuation coil versus the horizontal distance between the transmitter coil and the receiver antenna under the input voltage of 700 mV and their vertical distance at 2 cm. (**E**) Direction-controlled steering motion in a confined pool covered by a transparent acrylic sheet.

To realize wireless in situ monitoring using the SES system, we first need to verify whether the SES can move freely and smoothly in confined spaces. Here, we designed a circular channel enclosed by a transparent acrylic panel, forming a confined circular pipe. Predictably, a wired swimming system in this passage would quickly become immobilized due to wire entanglement even if the wire is very long and thin. However, our untethered SES can perform repeated close-loop swimming in this confined channel without wire interference (Fig. 3A and movie S2). For a two-lap swimming experiment, the angle change versus time shows a linear relationship, proving the stable and uniform motion of the SES (fig. S4). By replacing the diodes D_1_ and D_2_ on the swimmer with two light-emitting diodes (LEDs), the SES was readily tracked in darkness by flashing LED lights (movie S3). Figure 3B shows a four-lap circular motion with SES positions marked by bright LED dots for each circle.

Furthermore, we granted SES steering capability by extending it to an upgraded system with two tails (Fig. 3C). Symmetrical paired powered antennas and electromagnetic oscillation units were integrated to achieve directional control. The navigability arises from asymmetric actuation voltages on both sides by adjusting the transmitter coil position above the upgraded SES. At 2-cm vertical distance, the actuation voltage reaches 7.1 V at a 700-mV input when the transmitter coil and receiver antenna are coaxial with 0-cm horizontal distance. As the center distance increases, the actuation voltage gradually decreases to 0.06 V at 5 cm. The specific actuation voltage distribution is shown in Fig. 3D. Thus, positioning the RF coil to cover both receiver antennas activates both electromagnetic units for forward swimming. Covering just one antenna side induces a turning motion (Fig. 3E, fig. S5, and movie S4).

### Resonance design and optimized swimming performance

Inspired by the swimming style of mammals like dolphins and even humans that beat their caudal fins or limbs to generate continuous undulation for efficient propulsion (table S2), we designed a dolphin-style soft swimmer (*34*). The swimmer contains an electromagnetic actuation module and a soft tail fin. With current input, the 1200-turn coil produces a magnetic field that attracts the magnets below via Lorentz force (Fig. 1B and fig. S6). Under the aforementioned unilateral square-wave voltage, the magnet oscillates to flap the caudal fin up and down for fluid propulsion (Fig. 1C, fig. S7, and movie S5). Meanwhile, to ensure a steady swimming posture, we prepared a soft floatable foam body from aerogel and silicone mixtures (fig. S8 and movie S6). The layout was optimized by three-dimensional (3D) finite element analysis (FEA) to match the swimmer’s center of mass and centroid (fig. S9 and table S3). Thus, the wireless battery-free SES was fabricated through integrated design.

To enable effective actuation at low power, we designed an electromagnetic resonant structure. Such structures vibrate most intensely at resonance, so finding the resonant frequencies is key for fast locomotion (*35*–*37*). Added mass induced by the surrounding fluid can tune the vibration properties including resonance modes and frequencies (*38*). For the uniform soft tail, tail length between magnet center and fin end markedly affects added mass. We thus studied vibration versus tail length from 3 mm to 23 mm (fig. S10). These 0.6-mm-thick tails were soft and tough (fig. S11). Simulated swimming based on a 2D equivalent model shows that the swimmer indeed swim fastest at resonance (movie S7) (*39*).

**Fig. 4.**
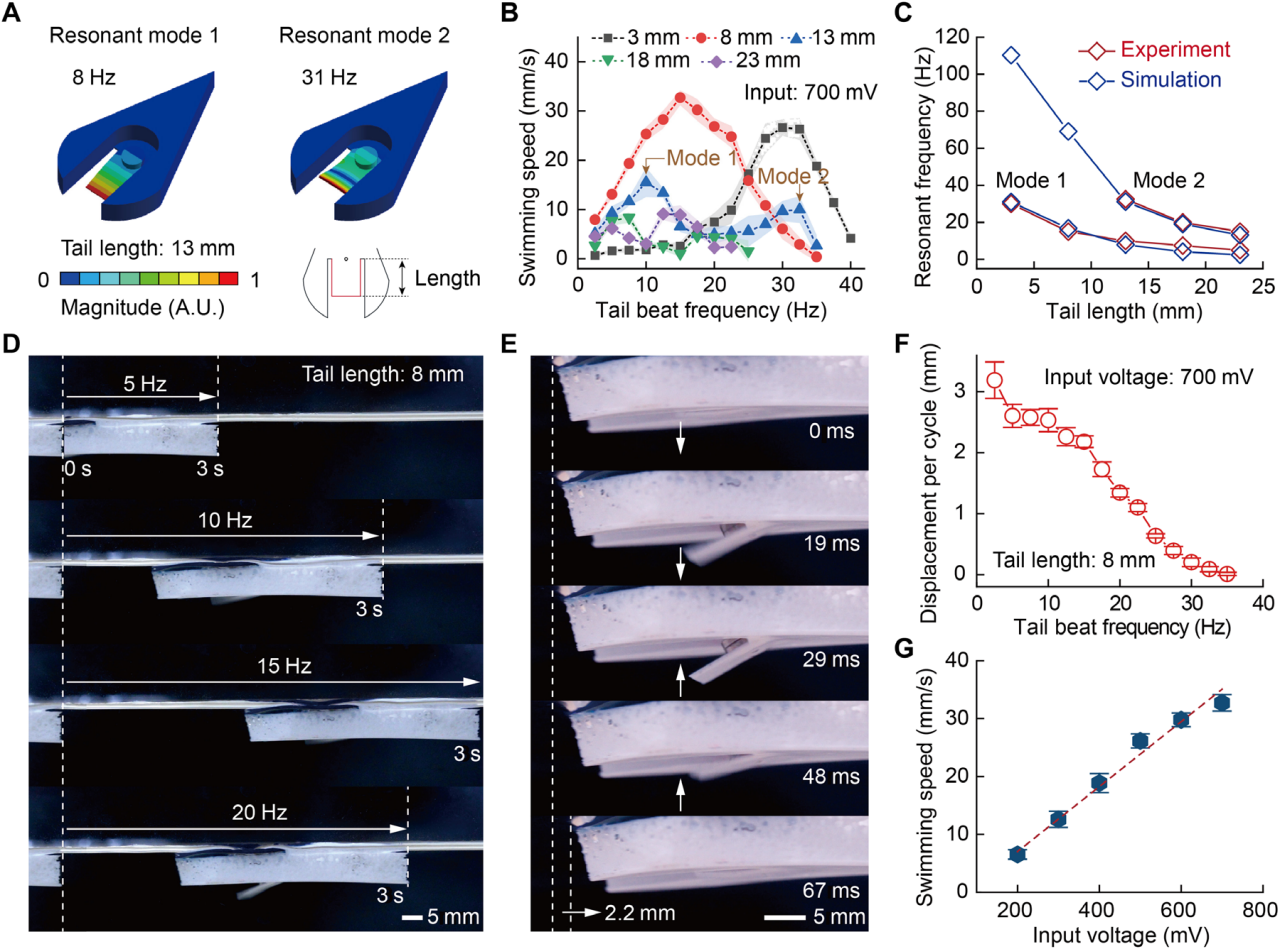
Resonance design and optimized swimming performance of the wireless SES. (**A**) Simulated resonant frequencies and vibration modes of the SES in water. The swimmer with a 13-mm-length tail shows two stimulated resonant modes at frequencies of 8 and 31 Hz. (**B**) Swimming speeds versus tail beat frequencies with different tail lengths. (**C**) Simulated and experimental resonant frequencies corresponding to resonant modes 1 and 2 for swimmers with different tail lengths. (**D**) Traveling distance of the 8-mm tail swimmer at different tail beat frequencies. A value of 15 Hz presents the fastest swimming speed. (**E**) Lateral view of the beating state of the soft tail fin during an actuation cycle. (**F**) Displacements per cycle versus tail beat frequencies of the 8-mm tail swimmer. (**G**) Relationship between swimming speed and input voltage.

We further performed 3D harmonic analyses to accurately model the vibration. The simulated first-, second-, and third-order resonant frequencies in air match well with the experimental values for the 23-mm tail (fig. S12), validating the simulation. Fluid-coupled analyses found resonant modes at 8 and 31.2 Hz, comparable to measured 10 and 32.5 Hz (Fig. 4A). The experimental swimming speed spectrum versus tail beat frequency agrees with simulation (Fig. 4C, fig. S13, and movie S8).

With a 2-cm transmitter-receiver distance, the 8-mm tail swimmer showed the fastest speed of 32.7 mm/s at 15-Hz first-order resonance and 700-mV input (Fig. 4, B and D, and movie S9), indicating that as low as 96 mW of power drives effective resonant propulsion. For pipe swimming (Fig. 2G), a 4-cm distance was also adopted to provide enough propulsion with only 13 mW of actuation power. Considering the fact that the swimmer swam faster in the water in its first-order resonance mode and the power consumption at different resonance modes was the same, we used the first-order resonance mode to actuate the swimmer. During the swim, the vibration system stayed subsurface, with the receiver antenna exposed above the water. Figure 4E and fig. S14 show the soft fin’s downward and upward beating in one cycle (movie S10). At 2.2-mm displacement per cycle, resonance gives the fastest swimming rather than 2.5 Hz with a maximum 2.6-mm displacement (Fig. 4F and fig. S15). Furthermore, higher voltage results in faster speed, demonstrating a linear relationship (Fig. 4G, fig. S16, and movie S11). When the actuation voltage across the actuation coil was reduced to 2.02 V, the swimming speed decreased to 6.5 mm/s. According to Fig. 2F, when increasing the actuation distance to 4 and 5 cm, the actuation voltages decreased to 2.59 and 1.75 V, respectively. Therefore, to ensure sufficiently high swimming speeds, the recommended maximum operational distance for the SES system is 4 cm.

### Integrated ionic and virus sensor

**Fig. 5.**
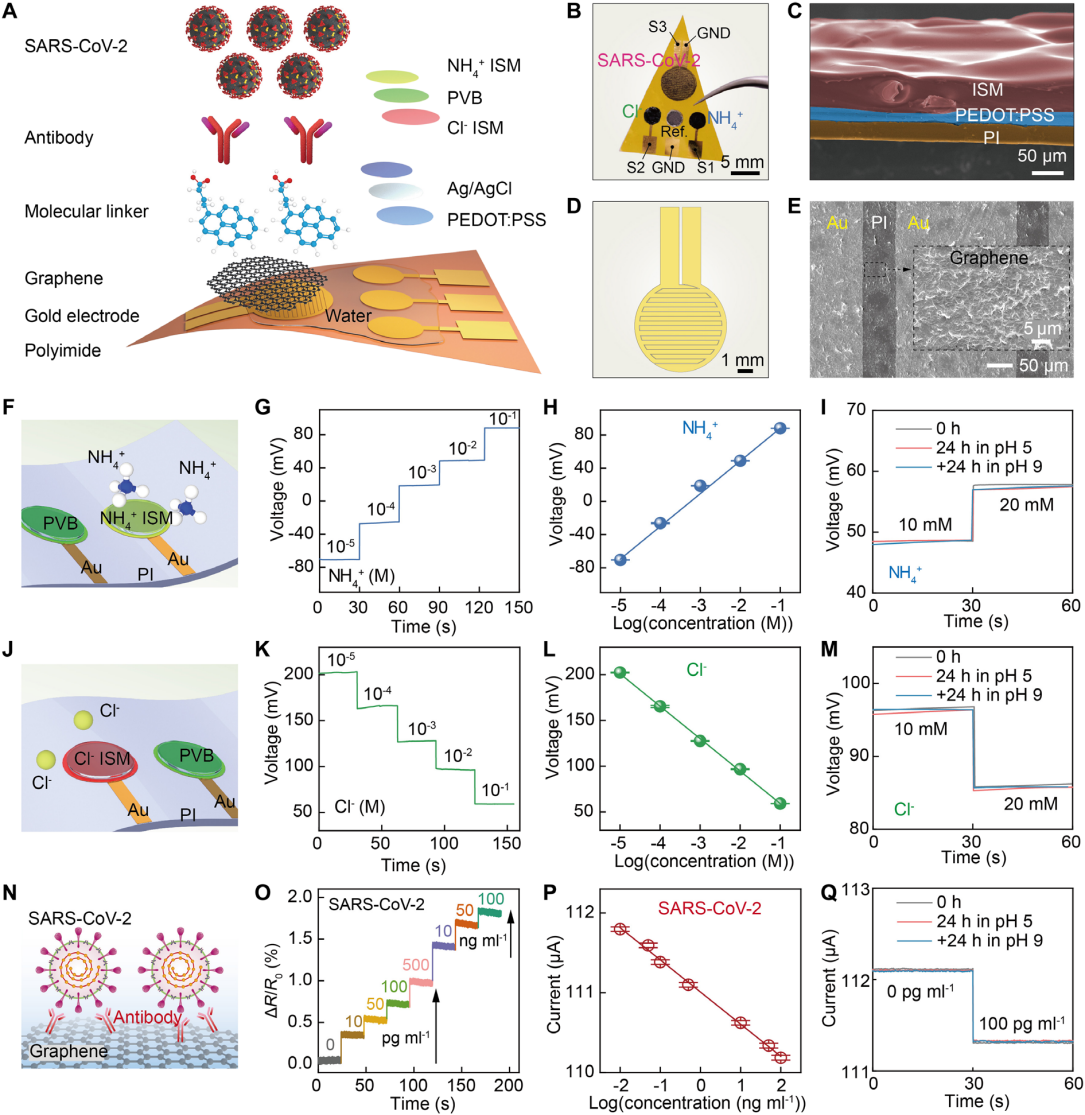
Integrated ionic and virus sensor. (**A**) Exploded-view schematic illustration of the integrated sensor with detection functions of NH_4_^+^/Cl^−^ and SARS-CoV-2 virus. (**B**) Photograph of the integrated sensor without encapsulation. (**C**) Cross-sectional scanning electron microscopy (SEM) image of the ion sensor with multilayer structures including PI, PEDOT:PSS, and ISM. (**D**) Design of an interdigital electrode for SARS-CoV-2 virus sensor. (**E**) SEM image of the SARS-CoV-2 sensor and the graphene that was sprayed onto an interdigital electrode. (**F**) Schematic diagram of NH_4_^+^ ion detection. (**G**) Voltage response of the NH_4_^+^ ion sensor at different NH_4_^+^ ion concentrations. (**H**) Linear relationship between NH_4_^+^ ion concentration and voltage response. (**I**) Anti-corrosion properties of NH_4_^+^ ion sensor. (**J**) Schematic diagram of Cl^−^ ion detection. (**K**) Voltage response of the Cl^−^ ion sensor at different Cl^−^ ion concentrations. (**L**) Linear relationship between Cl^−^ ion concentration and voltage response. (**M**) Anti-corrosion properties of Cl^−^ ion sensor. (**N**) Schematic diagram of the SARS-CoV-2 immune sensor. (**O**) Percentage change in impedance values under different concentrations of SARS-CoV-2 virus solutions. (**P**) Linear relationship between virus concentration and current values at an applied test voltage of 0.2 V. (**Q**) Anti-corrosion properties of SARS-CoV-2 immune sensor.

To endow the SES system with comprehensive monitoring capabilities, an integrated multifunctional sensor system is necessary. As illustrated in Fig. 5A, to construct the integrated sensors, a gold electrode pattern with three circular electrodes and an interdigital electrode on a 25-μm-thick PI film was used (Fig. 5B and fig. S17). To fabricate the chemical sensors, a poly(3,4-ethylenedioxythiophene) polystyrene sulfonate (PEDOT:PSS) layer was first electrodeposited on the two outer circular gold electrodes as the ion transducer. Ion-selective membrane (ISM) for ammonium ions (NH_4_^+^) and chlorine ions (Cl^−^) were then integrated on the PEDOT:PSS layer to form the functional electrodes (Fig. 5C and fig. S18). An Ag/AgCl layer was fabricated on the middle circular electrode and coated with a polyvinyl butyral (PVB) reference cocktail to form the reference electrode (*40*).

To fabricate the biosensor for severe acute respiratory syndrome coronavirus 2 (SARS-CoV-2) detection, a graphene dispersion layer was sprayed on the interdigital electrode to establish the basal electrode (Fig. 5, D and E). The 1-pyrenebutyric acid (PBA) molecular linker and SARS-CoV-2 spike S1 antibodies were then modified on the graphene surface. After blocking with bovine serum albumin (BSA), the uniformly distributed graphene-bound antibodies could precisely capture SARS-CoV-2 antigens with high specificity (fig. S19) (*29*, *41*).

For ion detection (Fig. 5, F and J), the sensor measured NH_4_^+^ and Cl^−^ concentrations based on the ion-induced electrochemical potential difference between the functional and reference electrodes (*20*, *27*, *28*). The NH_4_^+^ sensor exhibited good linearity from 10^−5^ M to 10^−1^ M in electrolyte solutions with naturally relevant concentrations (Fig. 5, G and H). Because of the ISM specificity, the sensor showed good selectivity and anti-interference ability without obvious responses to other ions or chemicals, such as Na^+^, Ca^2+^, Mg^2+^, and Cl^−^ (fig. S20A). Similarly, the Cl^−^ sensor demonstrated extensive sensing from 10^−5^ M to 10^−1^ M with good linearity and selectivity (Fig. 5, J to L, and fig. S20B).

For virus detection, the antibody binding to the SARS-CoV-2 spike protein altered the sensor impedance, enabling label-free detection of SARS-CoV-2 concentration (Fig. 5N). The label-free immune sensor also showed high sensitivity and linearity (Fig. 5, O and P). Owing to the interdigital electrode amplification and high graphene conductivity, SARS-CoV-2 concentrations as low as 10 pg/ml produced substantial impedance changes. The large graphene surface area and biocompatibility allowed sufficient antibody modification to achieve an extensive detection range from 10 pg/ml to 100 ng/ml. Antigen-antibody binding is typically irreversible (*42*). In actual use, when the sensor reaches its lifetime, we can replace a new sensor on the robot to process accurate detection again.

To ensure that the sensor can function normally in contaminated domestic water, we studied the stability of the device under extreme pH values. The sensor was soaked in the solutions of pH 5 and 9 for 24 hours in sequence, and then its performance was verified after each soak. As shown in Fig. 5 (I and M), after each soaking for 24 hours in acidic or alkaline solutions, the chemical sensors demonstrated almost the same sensing data as before soaking. For the virus sensors that were soaked in solution with pH 5 for 24 hours and in solutions with pH 5 and 9 in sequence for 48 hours, the sensors both maintain stable virus sensing capabilities (Fig. 5Q).

### Demonstrations of wireless monitoring in confined space via battery-free SES system

**Fig. 6.**
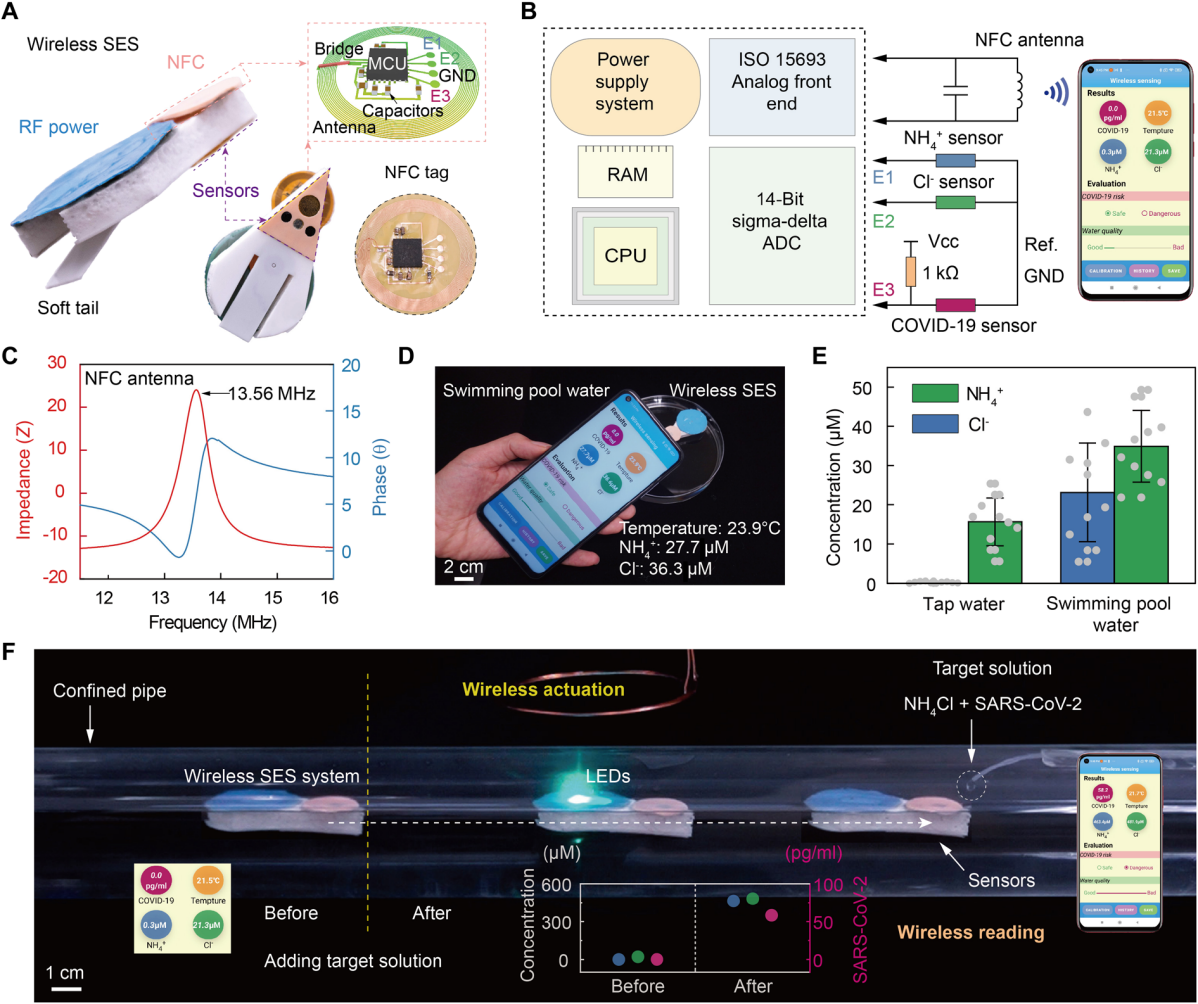
Wireless monitoring of ions and virus by the battery-free SES system. (**A**) Side and bottom view of the battery-free SES integrated with wireless powering and sensing modules. (**B**) Circuit logic diagram and smartphone interface for wireless sensing. (**C**) Operating resonance frequency of the NFC module. (**D**) Testing the swimming pool water via the SES system and a smartphone. (**E**) Ion concentrations of tap water and swimming pool water detected by the SES system. (**F**) Application demonstration: untethered actuation of the battery-free SES system and its wireless monitoring of ions/virus in a confined pipe.

Physically retrieving monitoring systems for data collection is challenging during exploration of confined spaces. To enable wireless readout, we integrated a lightweight, battery-free NFC module into the SES system. As illustrated in Figs. 5B and 6 (A and B), four ports including E1, E2, E3, and GND connected two ion sensors (S1, S2, and GND) and one virus sensor (S3 and GND) to the NFC chip (RF430FRL52H). A new sensor can be replaced after fully reaching its lifetime, while the electronic/NFC module can be used all the time without replacement. In addition, the NFC chip has a built-in temperature sensor to measure the core temperature of the chip. Since the chip’s working time is very short to generate heat, we regard this sensor temperature as the detected ambient temperature. The 13.56-MHz NFC circuit operated far from the 5.3-MHz resonant frequency of receiver antenna, preventing interference between the wireless components (Fig. 6C and fig. S21). The NFC allowed the microcontroller unit (MCU) to harvest energy from NFC-enabled smartphones through electromagnetic fields. It then digitized the sensor data via analog-to-digital conversion and transmitted the readings to the smartphone. For real-time data visualization, we developed a GUI on the smartphone to display temperature, water quality, and SARS-CoV-2 concentration measurements (Fig. 6B). Additionally, the smartphone could save the data and transfer it to a computer for further analysis.

As illustrated in Fig. 6A and fig. S22, the final wireless miniaturized SES system integrated electromagnetic resonance, battery-free wireless RF power, NFC data acquisition, and multifunctional sensors. To validate the monitoring capabilities of the SES system, we first assessed ion conditions in two typical water sources—swimming pool and tap water. For swimming pool water, NH_4_^+^ concentration is an important indicator of water quality, as its levels rise once water changes are not adequate or fecal contamination from swimmers happens. Outdoor swimming pool water samples were tested for NH_4_^+^ content. The previously determined NH_4_^+^ sensor calibration curve (Fig. 5H) programmed the NFC circuit for accurate measurements. By immersing the SES system in swimming pool water and scanning with a smartphone, the GUI could display the NH_4_^+^ level, as shown in Fig. 6D. For drinking tap water, Cl^−^ concentration indicates water quality since tap water derives from filtered, purified, and disinfected natural sources. Excessive residual hypochlorite or other disinfectants are unhealthy. As an example, laboratory tap water was tested and found to meet Hong Kong regulations. The GUI showed greatly higher NH_4_^+^ and Cl^−^ levels in swimming pool versus tap water (Fig. 4E).

Finally, we evaluated the SES system for detecting pathogens and ionic pollution in daily life pipelines using a simulated confined plastic pipe (Fig. 6F and movie S12). The fully wireless operation enabled free motion and positioning of the SES system within the pipe using only an external coil. In this monitoring experiment, SARS-CoV-2 was added as one detection target, considering its prevalence in recent years. First, pure tap water filled the confined pipe, leaving only a thin tube to add solutions. With the SES stationary at one end, the system was scanned by a smartphone and sensing data were read out as a comparative baseline. The GUI displayed 0.0 pg/ml SARS-CoV-2, 0.3 μM NH_4_^+^, and 21.3 μM Cl^−^ in the pure tap water (fig. S23). Then, 1 ml of target solution with 10 mM NH_4_Cl and 1 ng/ml SARS-CoV-2 spike protein was added and diffused. The system was then wirelessly actuated to move to the virus area. After 2 min to stabilize, rescanning displayed 58.2 pg/ml SARS-CoV-2 spike protein, 463.4 μM NH_4_^+^, and 481.9 μM Cl^−^, indicating effective detection of the added targets. These results demonstrate the SES system’s promise for monitoring confined spaces such as long domestic and industrial pipes. The water detection of ions and pathogens could help prevent diet-related diseases and viral infections.

## DISCUSSION

The results reported here demonstrate that a wireless, battery-free, miniaturized SES electronic monitoring system can serve as an effective platform for real-time wireless monitoring of physical (e.g., temperature), chemical (e.g., NH_4_^+^ and Cl^−^ ions), and biological (e.g., SARS-CoV-2 virus) parameters in confined environments such as pipelines. In such confined spaces, manual in situ sampling poses immense difficulties. Although mobile monitoring equipment enables in situ analyses, extracting this equipment whenever needed remains highly inconvenient. The SES system integrates fully wireless operation for both actuation and sensing data acquisition, allowing untethered mobility within confined areas without cables. This untethered functionality enables real-time in situ positioning and monitoring at any location inside confined spaces, eliminating the need to remove equipment for data extraction. Additionally, the SES system’s detection capabilities cover a variety of ion detection and pathogen detection for assessing water quality and viral contamination, noticeably advancing robotic monitoring capabilities and intelligence.

In this mobile electronic monitoring platform, an electromagnetic resonance swimmer was developed and implemented as the carrier, enabling the monitoring system to reach any position for on-demand detection. The resonance design allows the swimmer to achieve faster movement speeds, similar to previous sheet-shaped electromagnetic resonance soft robots (*43*) and piezoelectric-based thin soft robots (*44*). Simulations revealed that the resonant frequency of the swimmer differs in water versus air, resulting from variances in the fluid environments. This finding provides an important reference for future research on resonant soft swimmers.

An RF power supply was implemented to wirelessly actuate this SES only with a thin receiver antenna, advantageously avoiding increased size and weight from batteries and their necessary supporting circuits. The RF electromagnetic actuation enables a noncontact, short-range, wireless actuation manner but requires only a transmitter coil rather than a specialized multi-axis magnetic control system, optical tracking system, or multichannel air pump system (table S5). Furthermore, the SES system demonstrates multiple sensing capabilities that other robots do not present. The limited working distance of the system does impose application constraints in remote environments like deep-water exploration. However, the system shows capabilities in routine monitoring of open swimming pools and confined pipelines. Considering that remote exploration in rivers and oceans does not require miniaturization, future optimization could focus on integrating batteries and acoustic communication module to greatly enhance the operational distance (*45*). Moreover, integrating the lightweight and soft ion and pathogen sensing module used in the SES system onto the previously reported underwater robot can easily develop new underwater devices for detection in deep water areas (*46*, *47*). In addition, RF power would also be an important active source for in vivo applications in a wireless form. For example, using a magnetic soft robot (*31*, *48*) or an ingestible capsule device (*49*, *50*) as a carrier, the system can freely enter the body and perform the in vivo medical tasks, such as active electrical stimulation therapy.

Wireless signal reading is also necessary for in situ monitoring in confined spaces. In this study, the battery-free NFC integration, along with the smartphone interface, provides key advances in portability and data accessibility. Users can visualize real-time measurements by simply scanning the SES system, overcoming previous limitations requiring physical extraction of sensors. This on-demand readout considerably improves application convenience and rapid condition assessment. The noninvasive nature and simple smartphone interface make the system well suited for use by nonexperts during initial hazard evaluation. However, the limited energy supplied by smartphones restricts the readable distance of NFC tags to under 10 cm. Using an additional RF coil to power the NFC chip and incorporating an external reading circuit could further extend this range. For applications requiring maximized transmission range, wirelessly powering Bluetooth with RF represents an alternative solution, simultaneously improving data transmission distance while eliminating battery needs (*51*, *52*). However, Bluetooth’s power demands would proportionally reduce available power for actuation system. Thus, the optimal balance between NFC and RF-powered Bluetooth should be determined by carefully evaluating operational priorities, weighing requisite data transmission range against necessary actuation power for the intended application.

The presented platform demonstrates temperature, ion, and virus monitoring capabilities. All sensors operate based on electrical signals, enabling seamless integration with the entire electrical system. As different detection substances can assess various indicators, enhancing the detection functionality of this platform will rely on integrating novel sensors. In the future, new opportunities may come from incorporating various sensors for more parameter monitoring such as heavy metals for assessing industrial contaminants (*53*, *54*), *Escherichia coli* to determine fecal contamination of water (*55*, *56*), and gastric acids to monitor stomach health. Expanding the sensing capabilities will be key to enabling this monitoring platform to operate in more application scenarios.

## MATERIALS AND METHODS

### Fabrication of the circuit electrodes

Flexible patterned copper-clad polyimide films (PI, 12.5 μm and Cu, 18 μm) were used as the circuit electrode substrates for RF wireless power and NFC wireless sensing modules. Photolithography and wet etching processes enabled the required electrode patterns (fig. S2). Briefly, a thin copper-clad PI film was affixed onto a glass substrate using PDMS (10:1 weight ratio of pre-polymer and cross-link agent, Sylgard 184, Dow-Corning) and cured at 75°C for 20 min. Positive photoresist (AZ P4620, AZ Electronic Materials) was then spin-coated onto the copper surface at 3000 rpm for 30 s, soft-baked at 115°C for 5 min, and exposed to 350-nm ultraviolet (UV) light through a predetermined photomask. After development in the developer [AZ400K, volume ratio of 1:3 with deionized (DI) water] and post-baking at 115°C for 5 min, the sample was wet-etched in FeCl_3_ solution to remove the uncovered copper. Residual positive photoresist was subsequently removed using acetone, yielding the desired Cu electrode geometry. Finally, the flexible patterned films were isolated by laser cutting (ProtoLaser U4, LPKF Laser & Electronics). This optimized microfabrication approach allowed facile patterning of robust copper electrodes on thin PI substrates for integrating the wireless modules.

### Wireless circuit module

A 50-turn flexible copper coil with an inner diameter of 10 mm, outer diameter of 30 mm, linewidth of 100 μm, and thickness of 18 μm patterned on the PI film was fabricated to serve as the RF receiver antenna. A 6-pF capacitor was connected in parallel with the antenna to tune the resonant frequency to match that of the transmitter coil. Two diodes were connected in series in the same direction to the circuit to obtain a unidirectional voltage. Then, a 2.2-μF capacitor was connected in parallel to stabilize the unidirectional AC voltage at a high potential, providing steady and continuous energy. Finally, an 1200-turn actuation coil (inner diameter 3 mm, outer diameter 12 mm, linewidth 50 μm, resistance 260 ohms) was connected in parallel with the 2.2-μF regulator capacitor to actuate the SES forward. For wireless sensing, a flexible NFC circuit was fabricated with an RF430FRL152H chip (Texas Instruments) and matched capacitors and resistors, operating at 13.56 MHz. Four open ports enabled integration of two ion-selective biosensors and one virus biosensor.

### Dimension design of the SES monitoring system

The FEA software Ansys Mechanical was used to design the SES system’s dimensions to enable stable and smooth swimming motion by aligning the centroid and center of mass. The shapes and sizes of the support, soft tail, and integrated sensor were iteratively designed, and the placement arrangement of the wireless RF power module, NFC module, actuation coil, and magnet was optimized through multiple iterations, allowing precise matching of the centroid and center of mass.

### Fabrication of the SES monitoring system

The SES monitoring system comprised the following components: a soft tail, a magnet, an aerogel foam support, an actuation coil, an integrated sensor, a wireless RF power module, and an NFC module. The soft tail was fabricated by spin-coating PDMS pre-polymer and cross-linker at a 10:1 weight ratio at 300 rpm for 30 s, followed by curing and laser-cutting to form a patterned film with a thickness of 0.6 mm. A magnet with a diameter of 6 mm and thickness of 2 mm was affixed to the soft tail to enable electromagnetic induction oscillation. The aerogel foam was formed by mixing and curing PDMS (30:1) and aerogel particles at a weight ratio of 30:1. After cutting and engraving, a floatable support structure with a thickness of 6 mm was obtained. Finally, all components were bonded together using 10:1 PDMS and the SES system was attained after curing.

### FEA of dynamic simulations

3D harmonic response analyses were carried out using the commercial software Ansys Mechanical to determine the resonant frequencies of the SES with varying tail lengths. Mesh independence was verified to ensure computational accuracy, where the minimal element size was one-sixth of the soft tail thickness. Fixed support was applied to the support module. Simulations for the resonant frequencies of the SES in air were performed, and the results agreed well with experimental results. Further harmonic response analyses coupled with the surrounding fluid medium were conducted to determine the resonant frequency of the SES in water. The simulated resonant frequency in water agreed well with experimental results. Vibration modes of the SES were illustrated in fig. S13. All material properties used in the simulations were listed in table S4. Additionally, simulations of the swimming behavior of the SES in water were performed using a 2D equivalent model in COMSOL Multiphysics software. Results demonstrated that the excitation frequencies corresponding to the fastest swim speeds were highly consistent with the frequencies obtained from the harmonic response analyses simulated in Ansys (movie S6).

### Wireless actuation of the SES

A four-turn single-layer copper coil with an inner diameter of 60 mm, outer diameter of 66 mm, and linewidth of 0.8 mm served as the RF transmitter coil. A waveform generator triggered by an Arduino Leonardo board produced a 5.3-MHz sine wave voltage with low-frequency (e.g., 15 Hz) on-off switching modulation, which served as the input voltage. The input voltage was then amplified through an E&I 2100L RF power amplifier (Electronics & Innovation Ltd.) and loaded onto the transmitter coil. As the transmitter coil approached the SES, RF energy was wirelessly supplied to the receiver antenna. By matching the resonant frequency between the transmitter coil and receiver antenna at 5.3 MHz via adjusting capacitor C_1_, energy transmission to the receiver antenna was optimized. After the received power passed through two diodes, D_1_ and D_2_, acting as rectifiers, and a capacitor C_2_ acting as a stabilizer, a unilateral square-wave actuation voltage was produced. This actuation voltage was then loaded onto the actuation coil to produce an oscillating magnetic field. Under the periodic Lorentz force, the magnets on the soft tail generated a periodic oscillation. Thus, the SES swam forward in the water.

### Electromagnetic simulation for the coupling coefficient *k*

FEA was used to calculate the coupling coefficient *k* between the coaxial transmitter coil and receiver antenna. In the FEA simulation, a current excitation was applied and a spherical surface with a 1000-mm radius was adopted as a radiation boundary. The electromagnetic material parameters of copper and PI from the material library were used. The coupling coefficient was calculated by the equation $k=\frac{\Psi_{\text{RT}}}{I_{T}\sqrt{L_{T}L_{R}}}$ where Ψ_RT_ is the magnetic flux linkage, *I*_T_ is the current in the transmitter coil, *L*_T_ is the self-inductance of the transmitter coil, and *L*_R_ is the self-inductance of the receiver antenna.

### Fabrication of the gold electrode for integrated sensors

A sheet of 25-μm-thick PI film was attached on a quartz glass and sequentially cleaned by acetone, ethanol, and DI water. Then, 10-nm-thick chromium (Cr) and 180-nm-thick gold (Au) were then deposited on the PI film by electron-beam evaporation system (Explorer22, Denton Vacuum). Photolithographic patterning of the metal layers was performed as follows. The sample was spin-coated with a positive photoresist (AZ 5214, AZ Electronic Materials) at 3000 rpm for 30 s, followed by baking on a hot plate of 110°C for 2 min. The photoresist was then exposed to UV light through a photomask and developed in AZ 300MIF developer. After a post-exposure bake at 110°C for 3 min, the exposed gold was etched away in a I_2_/KI gold etchant solution, leaving the desired electrode pattern. Finally, the remaining photoresist was removed with acetone, resulting in patterned gold electrodes on the PI film composed of three circular electrodes and one interdigitated electrode.

### Fabrication of chemical ion sensor

To fabricate ISEs, the PEDOT:PSS layer was first electrodeposited on the two outer circular Au electrodes using a CHI 660E electrochemical workstation at a constant current of 0.2 mA/cm^2^ for 1800 s. The deposition solution was the mixed solution of 0.015 M 3,4-ethylenedioxythiophene (EDOT) and 0.1 M polystyrene sulfonate (NaPSS). Next, 5 μl of chloride ionophore I-Cocktail A (Sigma-Aldrich) and NH_4_^+^ selective membrane cocktail were dropped on the fabricated electrode to create the Cl^−^ and NH_4_^+^ selective electrode, respectively. The NH_4_^+^ selective membrane cocktail consisted of 1 mg NH_4_^+^ ionophore (nonactin), 33 mg of PVC (K-value 72-1), and 66 mg of bis(2-ethylehexyl) sebacate (DOS) dissolved in 660 μl of tetrahydrofuran. Then, an Ag/AgCl reference electrode was fabricated on the middle circular electrode using a screen-printing method with Ag/AgCl ink. This reference electrode was then coated with 2.5 μl of a PVB reference cocktail that was prepared by dissolving 79.1 mg of PVB, 50 mg of sodium chloride, 1 mg of block polymer PEO-PPO-PEO (F127), and 0.2 mg of carbon nanotubes in 1 ml of methanol. In this way, ISEs capable of detecting NH_4_^+^ and Cl^−^ were realized.

### Fabrication of SARS-CoV-2 virus sensor

Graphene dispersion (0.4 ml, 1 mg/ml) was spray-coated onto the interdigital gold electrode on a hot plate at 140°C. The sprayed graphene sample was treated with UV-ozone (UVO) cleaning in a Jelight 144AX-220 cleaner for 5 min and immersed in DI water for 12 hours to remove the sodium dodecyl benzene sulfonate surfactant. Next, 10 μl of PBA (5 mM in methanol) was linked to the graphene surface and rested in methanol atmosphere at room temperature for 3 hours. Following the sample washing with methanol and air dry, 5 μl of mixed solution of EDC [1-ethyl-3-(3-dimethylaminopropyl)carbodiimide; 0.4 M] and NHS (*N*-hydroxysuccinimide; 0.1 M) in MES (0.025 M) was added on graphene surface and rested for 1 hour. After washing the sample by DI water, SARS-CoV-2 spike S1 antibody (5 μl, 250 μg/ml; Sino Biological, catalog no. 40150-R007) was added onto the graphene and rested for 4 hours. Finally, the sensor was blocked with 2% BSA in 1× phosphate-buffered saline (PBS) buffer for 1 hour. Through this process, this sensor was functionalized to enable the detection of SARS-CoV-2 virus.

### Electrical characterization of the integrated sensors

The calibration of ion sensors was measured by PowerLab 16/35 (PL 3516, AD Instruments). Calibration solutions containing various concentrations of NH_4_Cl were prepared by doping a 10-ml solution with ammonium chloride. During calibration, the solutions were stirred continuously to quickly reach the dynamic equilibrium. A stable time of 120 s was used between each measurement step. For SARS-CoV-2 detection, 50 μl of spike protein (Sino Biological, catalog no. 40591-V08H) at each test concentration was added dropwise onto the graphene surface of the sensor. When the spike protein was bound with antibody (Sino Biological, catalog no. 40150-R007), impedance of the sensor would increase, which corresponded to a decrease in current at a constant applied voltage. To enable efficient antibody-antigen binding and current stabilization, measurements were performed for 10 min at 0.2 V using the CHI660E electrochemical workstation. Between each test, the sensor was rinsed with PBS buffer to remove the previous sample.

### NFC phone interface for wireless sensing

The mobile application was developed using Android Studio 2021.1. The ion-selective sensors generated analog voltage signals proportional to the ion concentrations. These analog signals were digitized by the built-in 14-bit analog-to-digital converter (ADC) of the RF430FRL152H microcontroller for further processing. Since the impedance of the SARS-CoV-2 sensor varied based on the number of viruses trapped, a fixed-value resistor was connected in series with the sensor. By applying a voltage across this series circuit and measuring the voltage drop across the fixed resistor using the ADC, the resistance of the SARS-CoV-2 sensor could be calculated using the principle of resistive voltage division. To read the sensor data, the phone’s NFC interface must be in close proximity to the NFC modules on the SES monitoring system. When the system’s NFC antenna entered electromagnetic field of the phone, the phone would transmit a read command to the NFC chip. Using the wirelessly harvested power from the phone’s electromagnetic field, the NFC chip responded to the read command and powered the external sensors. The NFC chip then coupled the sensor data to its antenna to transfer the data back to the phone. Smooth calibration curves relating voltage/impedance to ion/virus concentration were fitted for each sensor. The digitized ADC signals were converted into concentrations by substituting the values into the corresponding calibration curve equations. The app displayed the calculated concentration data and evaluated water quality or disease risk to provide an assessment to the user based on predefined concentration thresholds.

### Wireless monitoring of ions and virus in confined pipe by SES system

To demonstrate the application of the SES system for ion and SARS-CoV-2 monitoring in domestic water pipes, we constructed an experimental confined pipe containing tap water. A stationary SES equipped with integrated sensing modules and NFC was positioned at one end of the pipe. The biochemical composition of the tap water could be easily read by bringing a smartphone near the system’s NFC antenna. After acquiring initial baseline readings, 1 ml of solutions containing 10 mM NH_4_Cl and 1 ng/ml SARS-CoV-2 spike protein was introduced into the tap water from the opposite end of the pipe to simulate contamination. To evaluate the change in water composition, the SES system was wirelessly powered by an external RF coil to autonomously swim to the new sample location. After allowing 2 min for the sensors to stabilize, a second set of measurements was obtained, detecting the expected changes caused by the introduced solutions. This demonstration highlights the utility of a mobile intelligent system for monitoring water quality and detecting biochemical alterations in confined pipe systems.
