# Supplementary material for: Battery-free, wireless, and electricity-driven soft swimmer for water quality and virus monitoring
Source: Sci Adv. 2024 Jan 10;10(2):eadk6301. doi: 10.1126/sciadv.adk6301 (PMC10780888; doi:10.1126/sciadv.adk6301)
Supplement: Supplementary file 1 — Figs. S1 to S23 Tables S1 to S5 Legends for movies S1 to S12 [file sciadv.adk6301_sm.pdf]

Science Advances
AAAS

Supplementary Materials for

Battery-free, wireless, and electricity-driven soft swimmer for water quality and virus monitoring

Dengfeng Li *et al.*

Corresponding author: Lingqian Chang, lingqianchang@buaa.edu.cn; Zhaoqian Xie, zxie@dlut.edu.cn; Xinge Yu, xingeyu@cityu.edu.hk

Sci. Adv. **10**, eadk6301 (2024)
DOI: 10.1126/sciadv.adk6301

The PDF file includes:

Figs. S1 to S23
Tables S1 to S5
Legends for movies S1 to S12

Other Supplementary Material for this manuscript includes the following:

Movies S1 to S12

Supplementary Figures

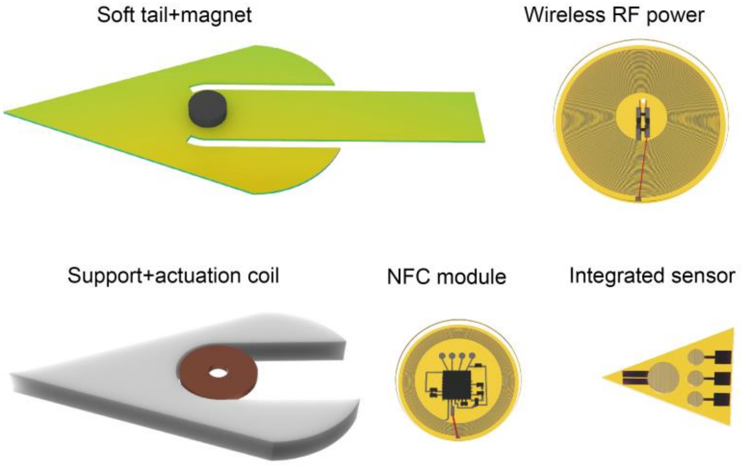

Fig. S1. Components of the wireless soft electromagnetic swimmer (SES) monitoring system.

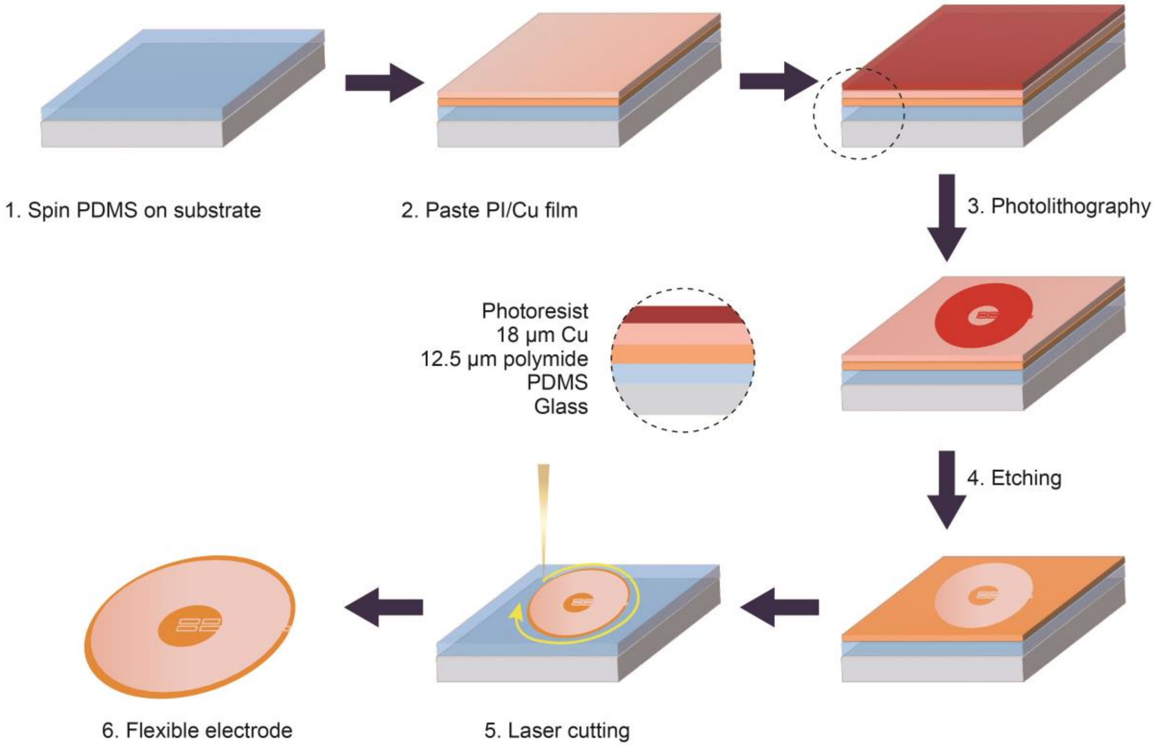

Fig. S2. Fabrication process of the flexible circuit copper electrode. Photolithography and wet etching are used to pattern 18-μm-thick copper electrodes on a 12.5-μm-thick polyimide substrate.

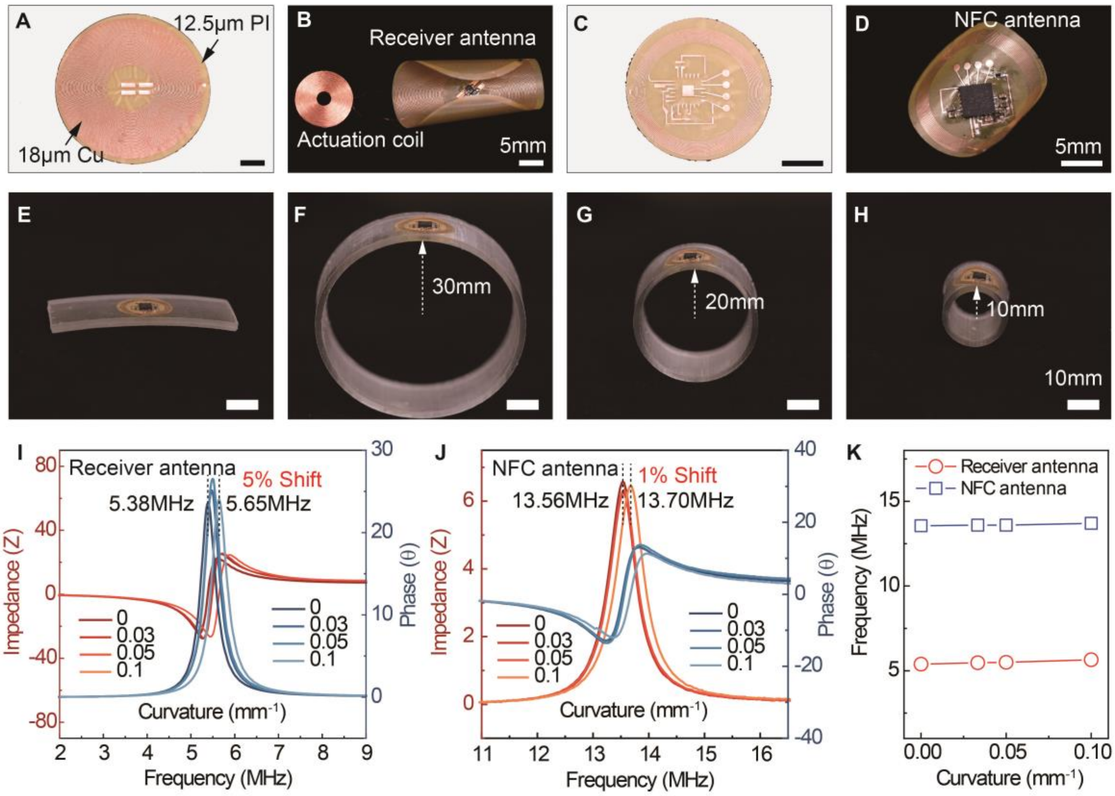

Fig. S3. Flexibility characterization of wireless power and communication modules. (A-D) Flexible receiver and NFC electrodes. **(E-H)** Modules on flat and curved surfaces. **(I-K)** Small resonant frequency drifts demonstrate stable wireless operation during bending.

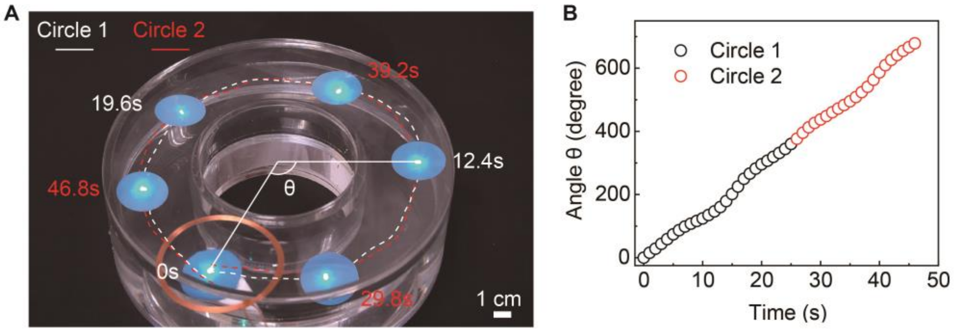

Fig. S4. Wireless SES' navigation in a closed-loop pipe. (A) The swimmer completes two laps around the circular channel through untethered, multi-turn steering. **(B)** The moving angles versus the time during the two laps.

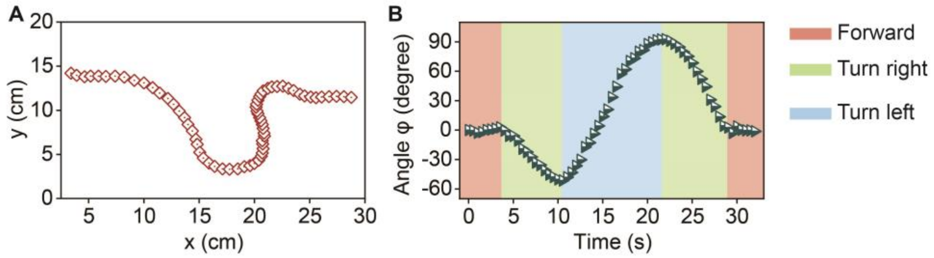

Fig. S5. Controllable path steering of the upgraded SES in confined spaces. (A) The coordinate location and steering path. **(B)** The direction steering during the motion. When the RF coil was directly above the two receiver antennas, the swimmer swam forward. When the RF coil was above one of the antennas, the swimmer turned to the left and to the right.

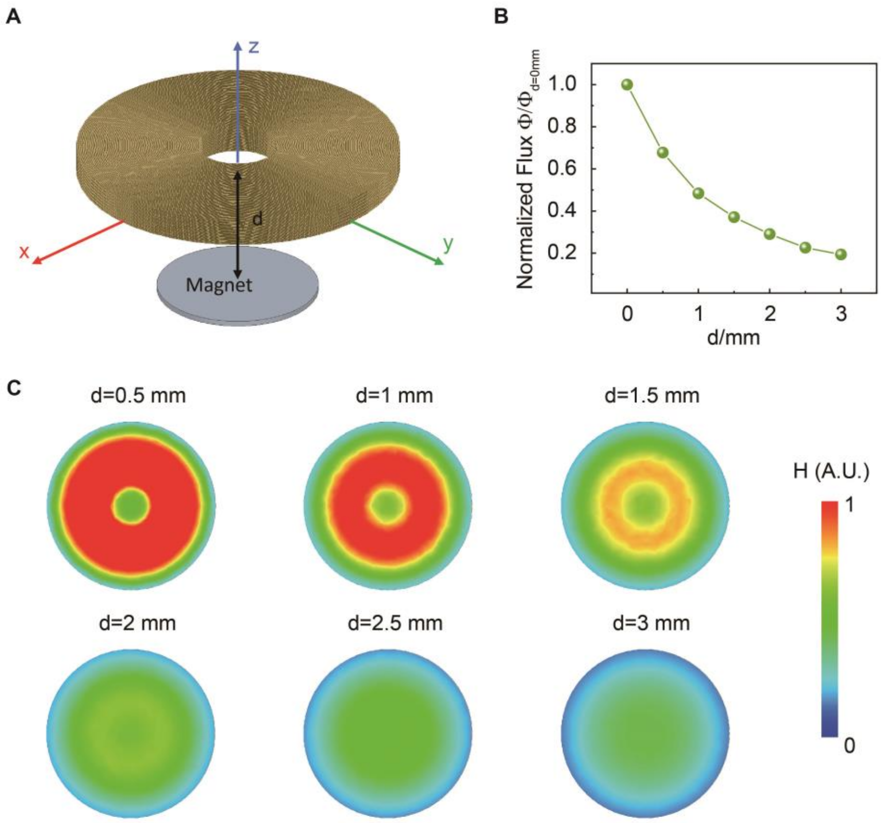

Fig. S6. Actuation coil magnetic field simulation. (A) Position of the magnets in relation to the actuation coil. **(B)** Plot of normalized flux versus distance between magnet and coil. **(C)** FEA simulation results of the actuation coil's magnetic field strength distribution at different distances.

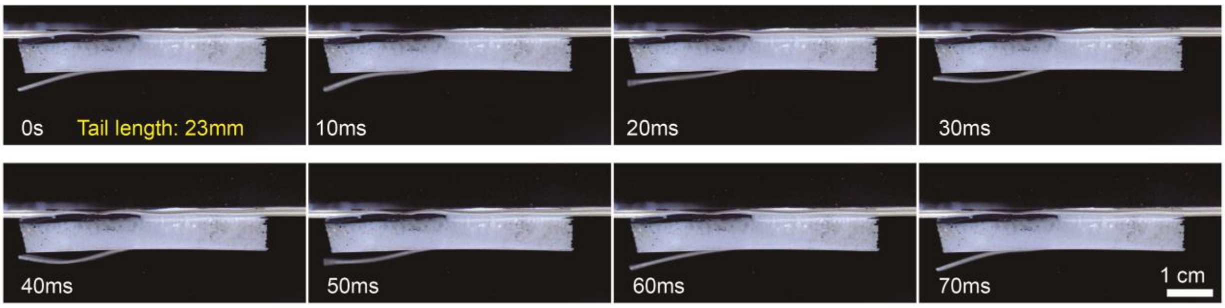

Fig. S7. Continuous tail undulation of SES in water for efficient swimming.

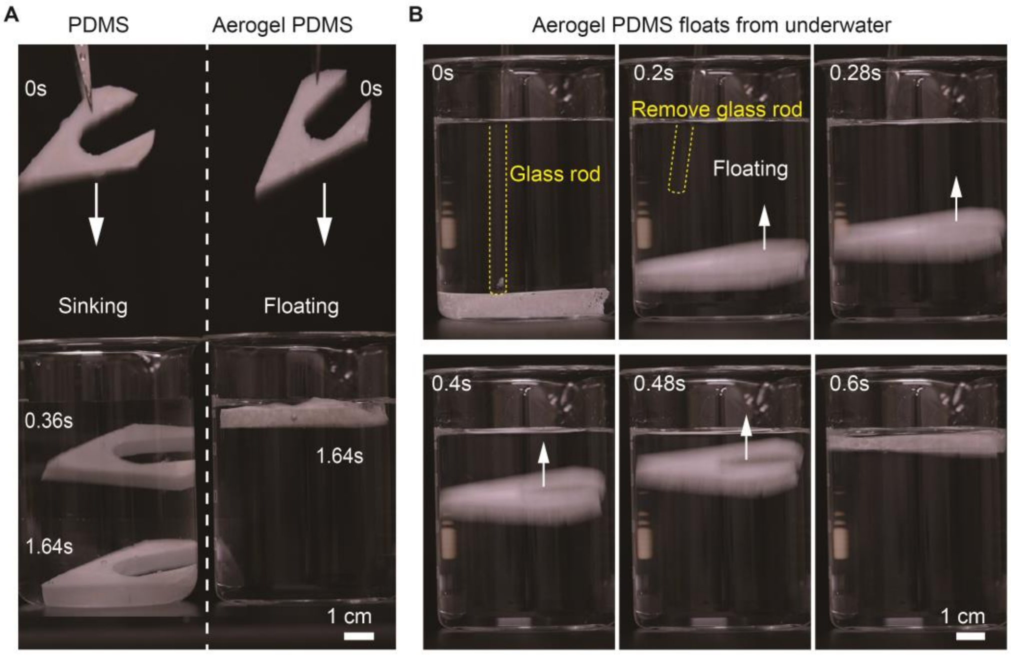

Fig. S8. Floating performance of the aerogel silicone foam. (A) A pure PDMS support and an aerogel PDMS foam support fall into the water. The pure PDMS support sinks to the bottom, yet the aerogel PDMS foam support floats. **(B)** The aerogel silicone foam support floats from the bottom to the surface of the water.

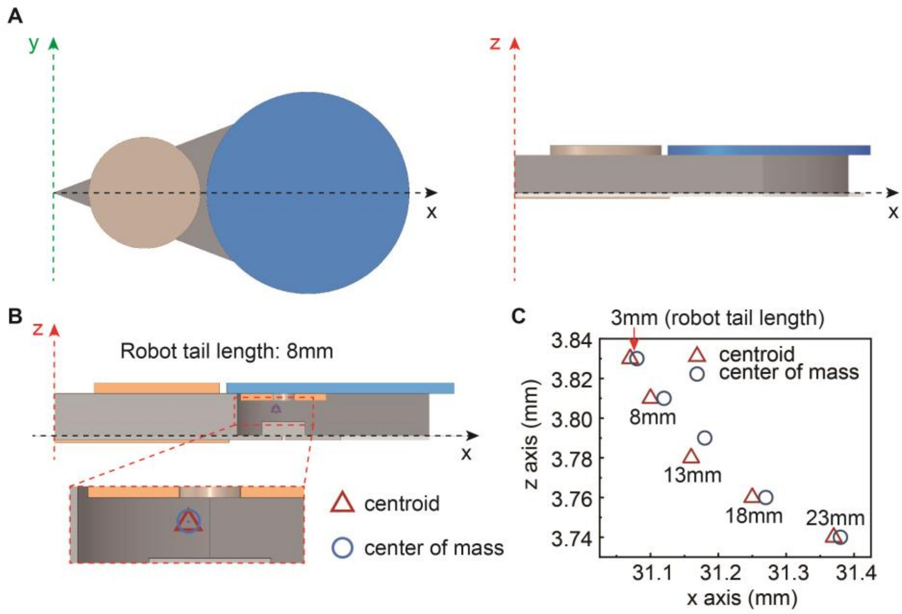

Fig. S9. Centroid and center of mass simulations. (A) Coordinate axes applied on the SES system from the top view and side views. **(B)** Centroid and center of mass positions for 8 mm tail SES system. **(C)** Centroid and center of mass positions for varied tail lengths.

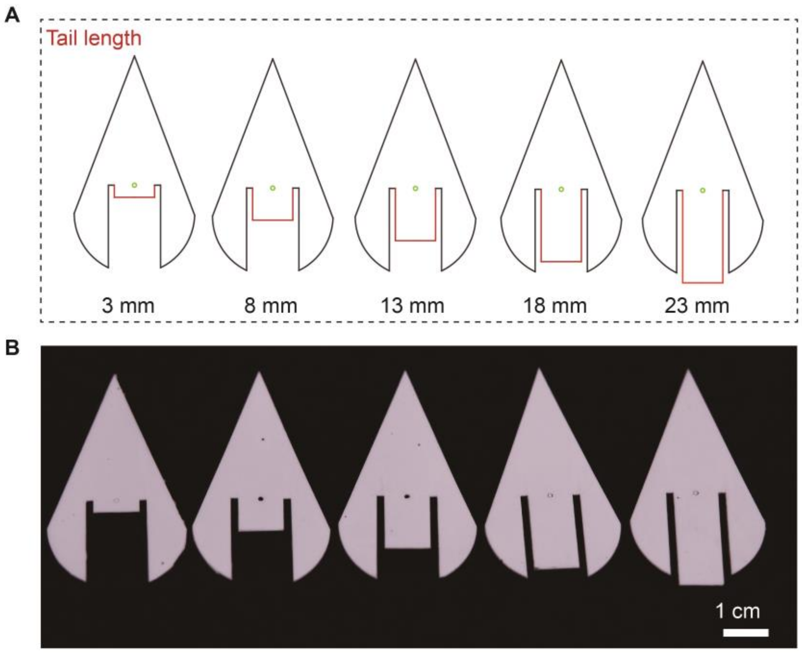

Fig. S10. Soft tails with different lengths. (A) Designs of tail lengths of 3 mm, 8 mm, 13 mm, 18 mm and 23 mm. Tail length is defined as the distance between the magnet center and the tail end. **(B)** Photograph of the fabricated tails.

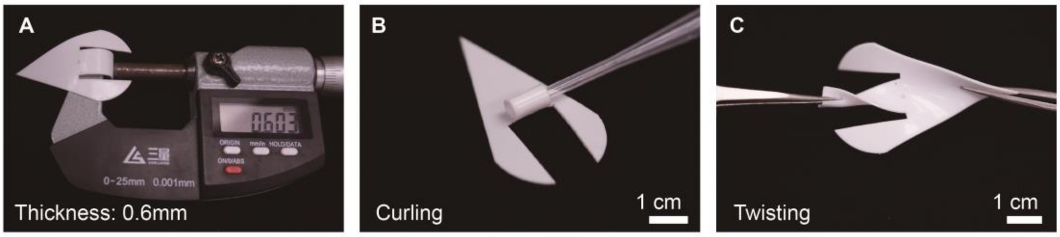

Fig. S11. Tail thickness and flexibility. (A) The swimmer tail thickness is 0.6mm. **(B)** Curling the swimmer tail with a round straw. **(C)** Twisting the swimmer tail.

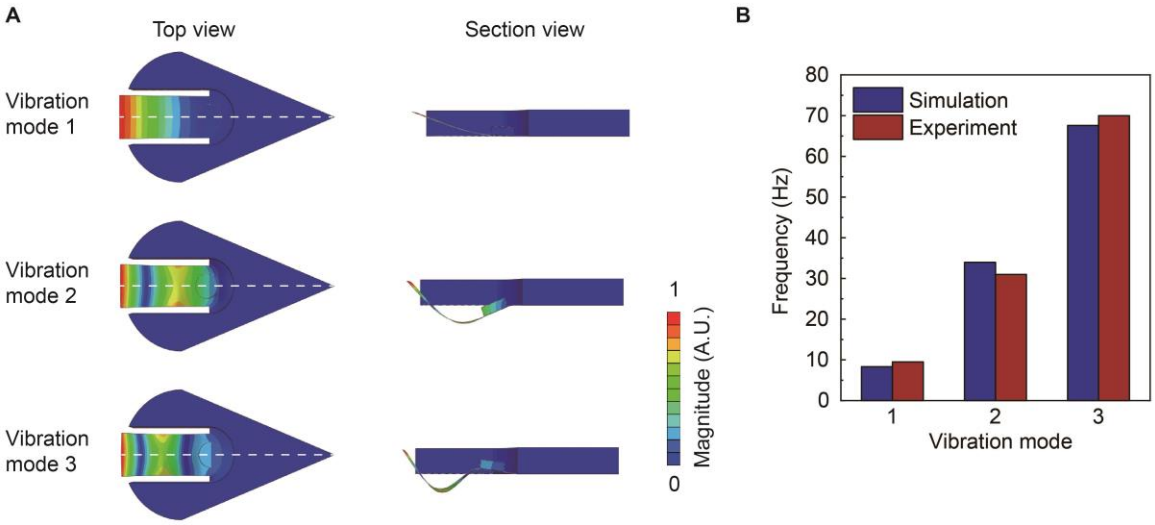

Fig. S12. FEA simulation analysis of the SES' resonant frequencies and vibration modes in air. (A) Schematic of the vibration modes' top view (left) and section view (right) along the white dashed line. **(B)** Resonant frequencies corresponding to vibration mode 1-3.

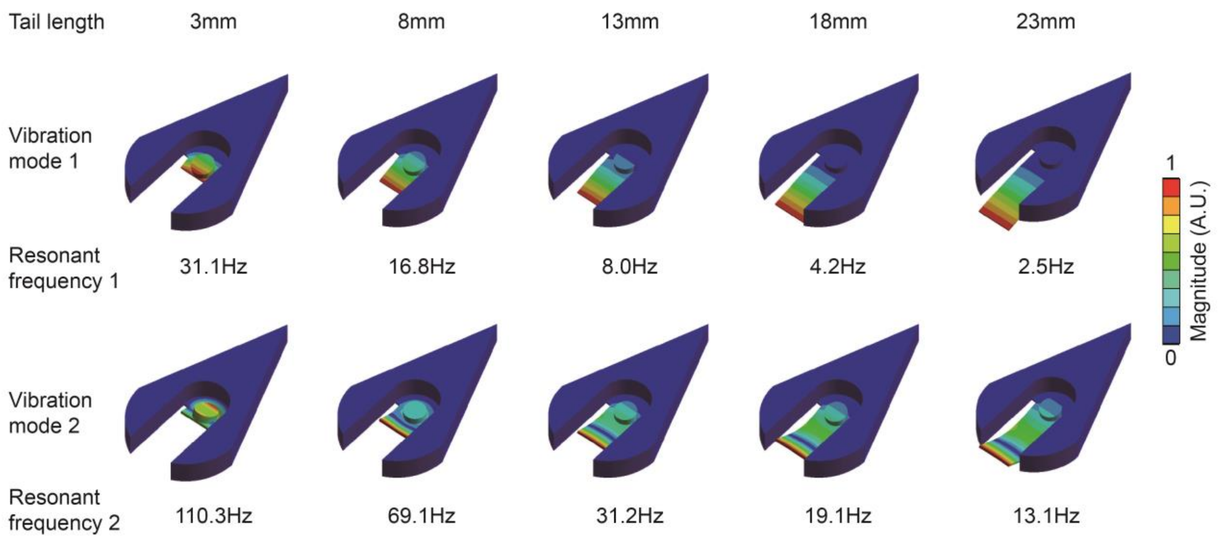

Fig. S13. FEA simulation analysis of the SES' resonant frequencies and vibration modes in water. Schematic of the resonant mode 1 (top) and resonant 2 (bottom) for the swimmer with different tail length.

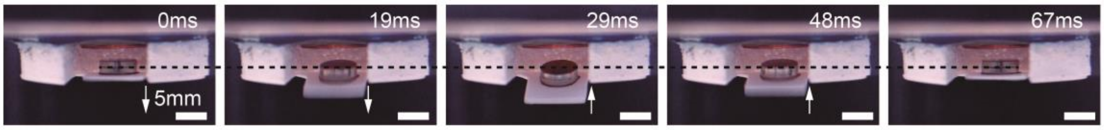

Fig. S14. Rear view of tail beating behavior during one actuation cycle.

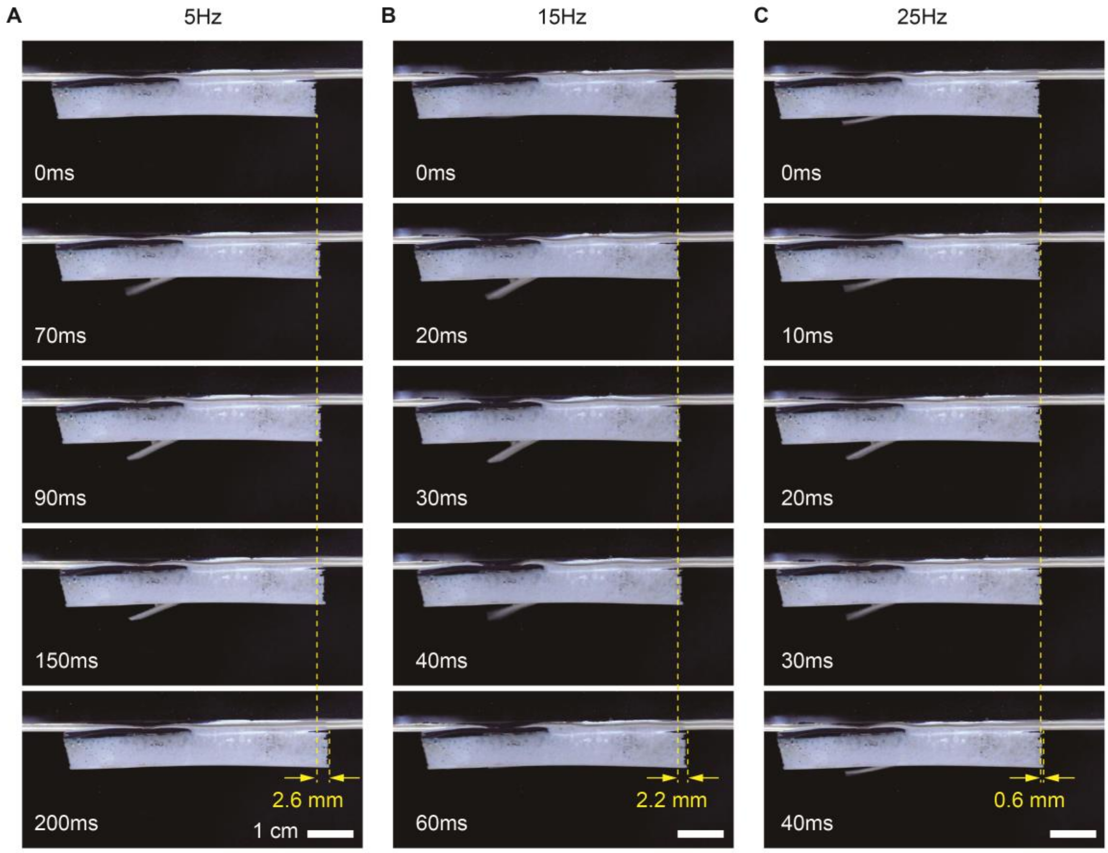

Fig. S15. Displacements per cycle of the 8 mm tail swimmer at different actuation frequencies. (A) 2.6 mm displacement at 5 Hz**. (B)** 2.2 mm displacement at 15 Hz**. (C)** 0.6 mm displacement at 25 Hz.

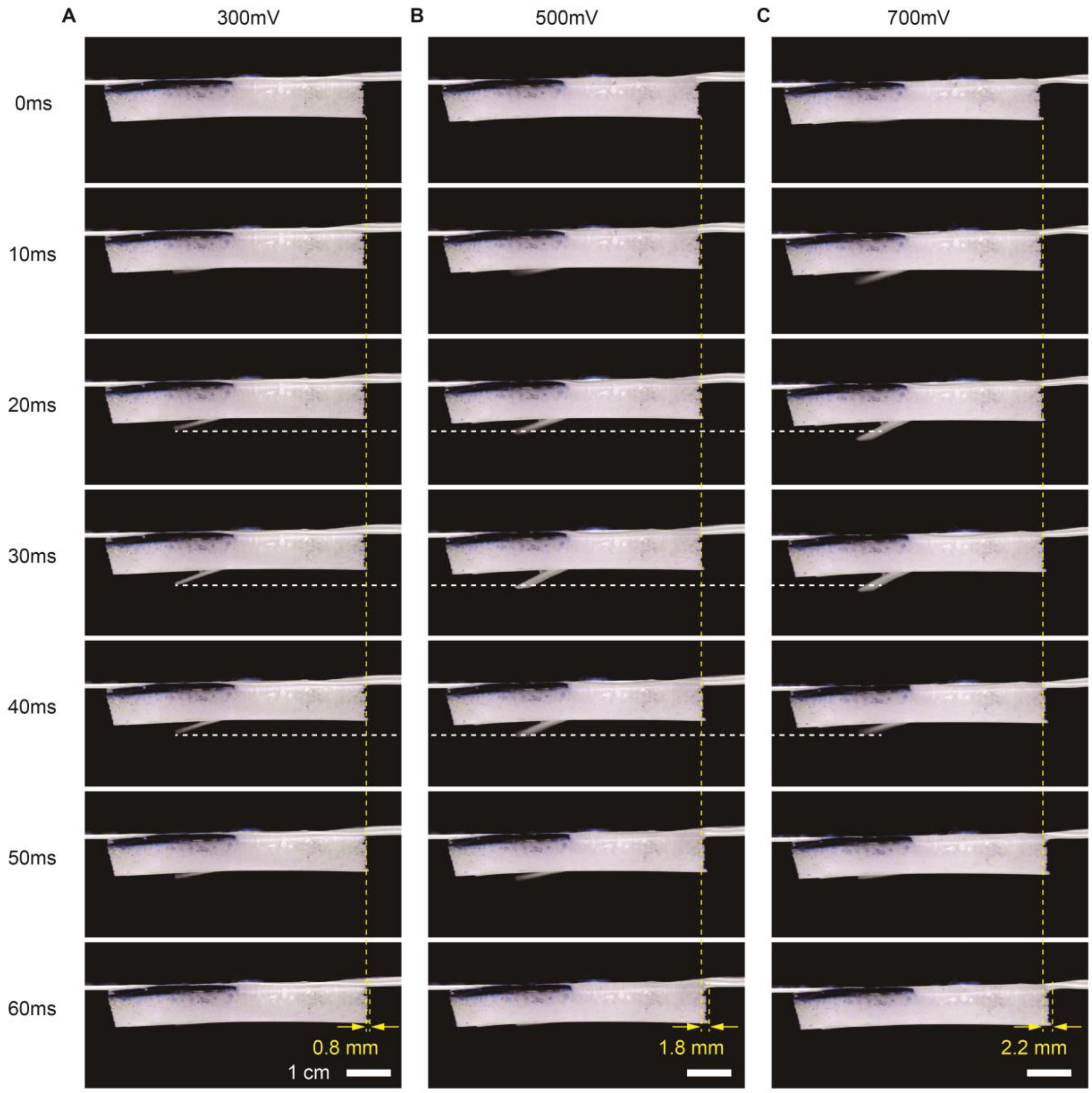

Fig. S16. Displacement per cycle versus input voltage for an 8mm tail swimmer at 15Hz. (A) 0.8 mm displacement per cycle at 300mV input voltage. **(B)** 1.8 mm displacement per cycle at 500mV input voltage. **(C)** 2.2 mm displacement per cycle at 700mV input voltage.

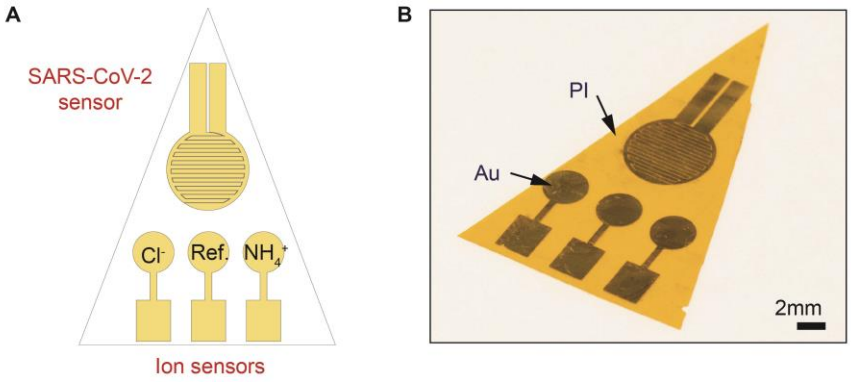

Fig. S17. Integrated sensor design. (A) Design of the integrated sensor with a SARS-CoV-2 senor, a Cl^- senor and a NH_4^+ sensor. **(B)** Photograph of the fabricated sensor electrodes.

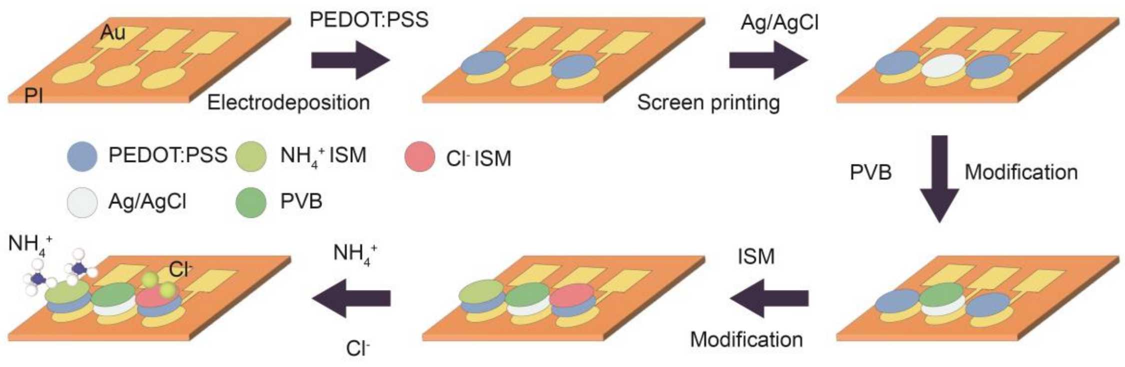

Fig. S18. Flow chart of ions sensor fabrication process.

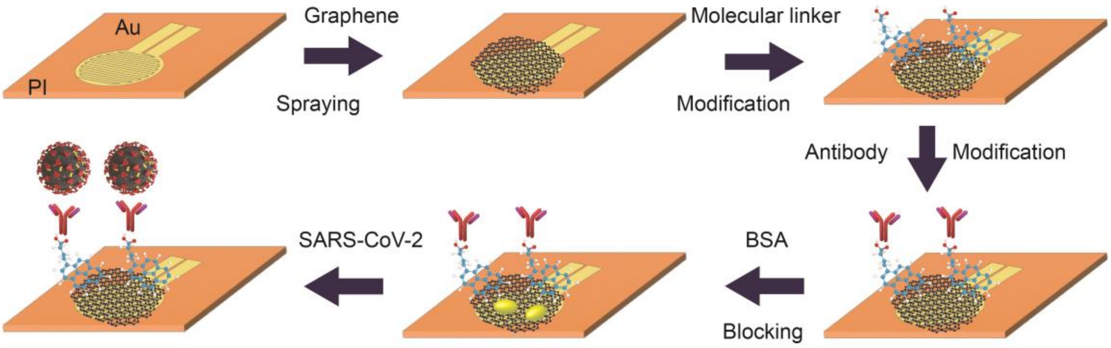

Fig. S19. Flow chart of SARS-CoV-2 sensor fabrication process.

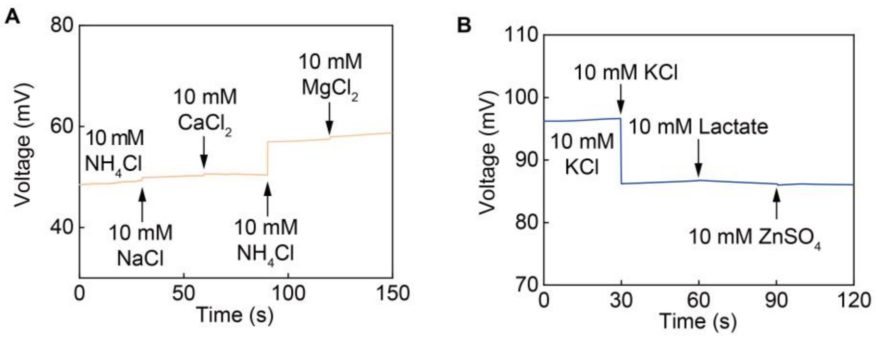

Fig. S20. Ion sensor selectivity testing. (A) Selectivity test of NH_4^+ ion sensor, which did not respond to Na^+, Cl^-, Ca^{2+} and Mg^{2+} ions. **(B)** Selectivity test of Cl^- ion sensor, which did not respond to K^+, Lactate, Zn^{2+} and SO_4^{2-} ions. Low responses to non-target ions demonstrate selectivity.

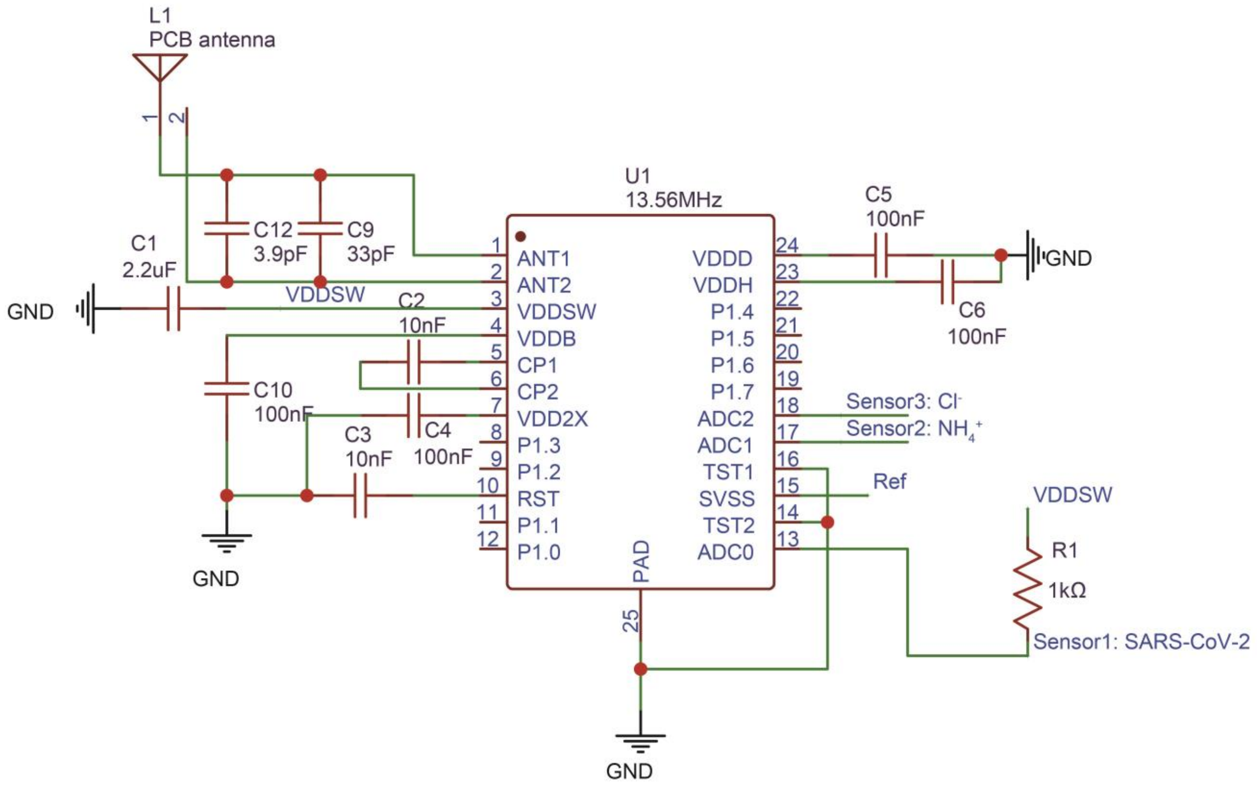

Fig. S21. Circuit diagram of the NFC module.

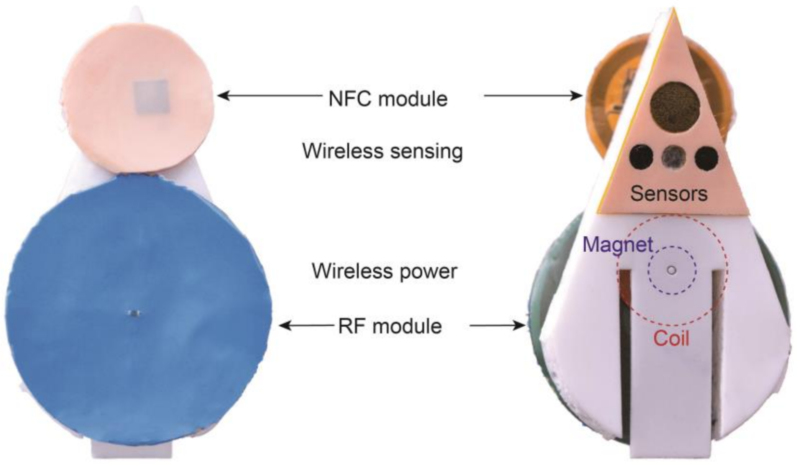

Fig. S22. Front- and back-view of the SES monitoring system.

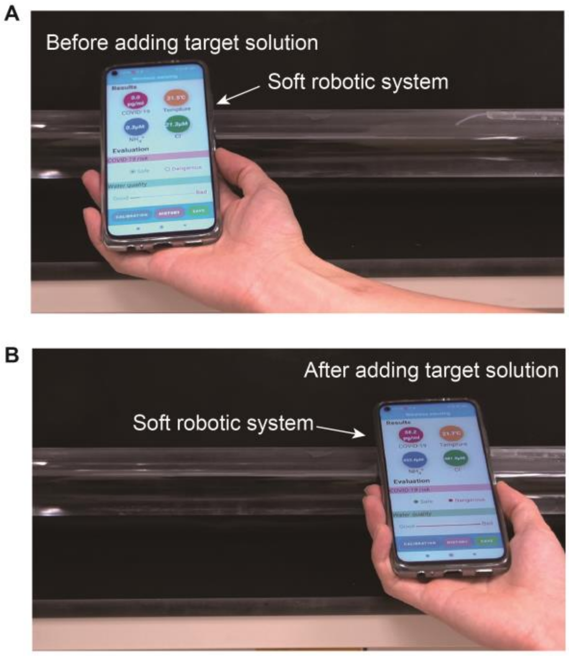

Fig. S23. Monitoring results displayed in GUI for target solution detection in a confined pipe by using SES system. (A) Detection results before dropping the target solution of NH_4Cl and SARS-CoV-2 spike protein. **(B)** Detection results after dropping the target solution and actuating the SES in the dropping position.

Supplementary Tables

Table S1: Summary of the mass and dimensions of individual SES system accessories.

SES accessories	Mass (g)	Length (mm)	Width (mm)	Thickness (mm)
Soft tail	0.425	52.7~55.2	29.8	0.6
Magnet	0.498	6	6	2
Aerogel silicon support	2.330	52.7	29.8	6
Coil	0.513	12	12	1
Wireless RF power module	1.134	32	32	1.5
NFC module	0.337	17.6	17.6	1.5
Integrated sensor	0.073	24.5	18.6	0.35

Table S2: Information summary of SES in this work and animals that swim in a similar manner.

Names	Mass (kg)	Length (m)	Swimming speed (km/h)	Swimming mode	Swimming posture
Blue whale	150,000 ~180,000	23~24	20~50	Tail fins beat up and down	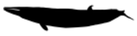
Bottlenose dolphin	150~650	2~4	9.7~18	Tail fins beat up and down	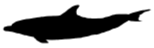
Enhydra lutris	30~50	1~1.4	10~15	Tail beats up and down, hind limbs paddle water backwards	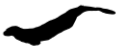
Dugong	300~500	2.6~3.3	10~20	Y-shaped tail fins beat up and down	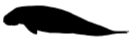
Swimmer	60~70	1.7~2	3~5	Legs beat up and down	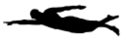
Diver	70~80	1.7~2	3.6~9	Legs beat up and down	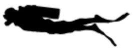
This work	0.00531	0.0527 ~0.0552	0.118	Tail beats up and down	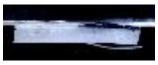

Table S3: Coordinates of the centroid and center of mass for the SES system.

**Tail length**	**3mm**	**8mm**	**13mm**	**18mm**	**23mm**
Centroid	(31.07, 3.83)	(31.10, 3.81)	(31.16, 3.78)	(31.25, 3.76)	(31.37, 3.74)
Center of mass	(31.08, 3.83)	(31.12, 3.81)	(31.18, 3.79)	(31.27, 3.76)	(31.38, 3.74)

Table S4: Material parameters used in the dynamic simulation**.**

**Materials/Parameters**	**Density** **ρ (kg/m^3)**	**Young's Modulus** **E (MPa)**	**Poisson's Ratio**	**Speed of Sound** ***v* (m/s)**
Aerogel silicone	660	3	0.5	/
Magnet	7620	1.6×10^5	0.3	/
Silicone B	960	1.5	0.5	/
Water	998.2	/	/	1482.1

Table S5: Comparison with other existing actuation approaches.

Reference	Actuation mechanism	Connection	Control system	Sensing ability
(*31*)	Magnetic	Wireless	Multi-axis magnetic control system, programmable control	/
(*32*)	Optical	Wireless	High-intensity laser, optical tracking system	/
(*33*)	Pneumatic	Wired	Multi-channel air pump system	/
This work	Electromagnetic	Wireless	A coil, an RF amplifier	Physical, chemical, biology

Reference are listed in the main text

Supplementary Movies

Movie S1: Positioning the SES system in opaque tubes by using an NFC transceiver to scan the tube.

Movie S2: Repeated close-loop swimming of the SES in a confined channel without wire interference.

Movie S3: Untethered four-lap circular swimming around a confined channel in darkness with LEDs tracking.

Movie S4: Directionally controllable steering motion in a confined pool.

Movie S5: Continuous tail undulation for efficient swimming.

Movie S6: Floating demonstration of the aerogel silicone foam.

Movie S7: Simulated swimming behavior of the SES at resonance.

Movie S8: Simulated resonant modes of the SES in water versus tail length.

Movie S9: Swimming performance of the 8 mm tail SES under different actuation frequencies.

Movie S10: Lateral and rear view of beating behavior of the SES' tail fin.

Movie S11: Swimming speed of the 8 mm tail SES versus input voltage.

Movie S12: Application demonstration: untethered actuation of the battery-free SES system and its wireless monitoring of ions/virus in a confined pipe.
